# Precision Oncology: Current Landscape, Emerging Trends, Challenges, and Future Perspectives

**DOI:** 10.3390/cells14221804

**Published:** 2025-11-17

**Authors:** Diane Qiao, Richard C. Wang, Zhixiang Wang

**Affiliations:** 1 Faculty of Medicine and Dentistry, College of Health Sciences, University of Alberta, Edmonton, AB T6G 2R3, Canada; dq@ualberta.ca; 2 UCSF School of Medicine, University of California, San Francisco, CA 94143, USA; richard.wang2@ucsf.edu; 3 Department of Medical Genetics, Faculty of Medicine and Dentistry, College of Health Sciences, University of Alberta, Edmonton, AB T6G 2H7, Canada

**Keywords:** precision medicine, precision oncology, subtyping, stratification, multiomics, targeted therapy, single-cell spatial omics, liquid biopsy, cancer initiation, tumor heterogeneity, tumor microenvironment

## Abstract

**Highlights:**

- This review summarizes the development, current state, challenges, and future outlook of precision oncology.
- It introduces technologies such as single-cell spatial multiomics, patient-derived tumor organoids, liquid biopsy, non-invasive imaging, and artificial intelligence.
- It reviews key concepts including tumor initiation, heterogeneity, and the tumor microenvironment.
- It emphasizes pan-cancer stratification and agnostic therapies as paradigm shifts.
- It highlights clinical applications span prevention, diagnosis, and treatment and its implementation challenges include infrastructure, education, costs, policy, and regulation.

**Abstract:**

Precision oncology is broadly defined as cancer prevention, diagnosis, and treatment specifically tailored to the patient based on his/her genetics and molecular profile. In simple terms, the goal of precision medicine is to deliver the right cancer treatment to the right patient, at the right dose, at the right time. Precision oncology is the most studied and widely applied subarea of precision medicine. Now, precision oncology has expanded to include modern technology (big data, single-cell spatial multiomics, molecular imaging, liquid biopsy, CRISPR gene editing, stem cells, organoids), a deeper understanding of cancer biology (driver cancer genes, single nucleotide polymorphism, cancer initiation, intratumor heterogeneity, tumor microenvironment ecosystem, pan-cancer), cancer stratification (subtyping of traditionally defined cancer types and pan-cancer re-classification based on shared properties across traditionally defined cancer types), clinical applications (cancer prevention, early detection, diagnosis, targeted therapy, minimal residual disease monitoring, managing drug resistance), lifestyle changes (physical activity, smoking, alcohol consumption, sunscreen), cost management, public policy, and more. Despite being the most developed area in precision medicine, precision oncology is still in its early stages and faces multiple challenges that need to be overcome for its successful implementation. In this review, we examine the history, development, and future directions of precision oncology by focusing on emerging technology, novel concepts and principles, molecular cancer stratification, and clinical applications.

## 1. Introduction

Precision oncology refers to the application of precision medicine in cancer treatment. It is the leading driver behind advances in precision medicine and represents its most researched and commonly used field.

Precision oncology refers to the use of broad molecular tumor characterization with the aim of personalized therapeutic management. In simple terms, the goal of precision medicine is to deliver the right cancer treatment to the right patient, at the right dose, at the right time.

The term ‘precision medicine’ first gained prominence after a publication by the US National Research Council in 2011 [1]. Four years later, precision medicine has become a term that symbolizes the new age of medicine following the launch of the national Precision Medicine Initiative by then President Obama in the United States in 2015 [2].

However, the use of molecular characterization of an individual patient’s tumor in routine oncologic practice roughly began 20 years ago, especially following the publication of the first draft of the Human Genome Project in 2003. Moreover, the idea and practice of precision medicine/oncology had even earlier origins, beginning in the 1980s and 1990s [2,3].

The early development of precision oncology was driven by a desire to move beyond blanket treatments for patients to a more refined, efficient, and patient-centred approach. To achieve this goal, three essential objectives must be met. First, the cancer needs to be stratified into various subtypes. Second, there must be tailored treatment available for each specific subtype. Third, comprehensive molecular profiling of each individual patient must be generated. When all three objectives are met, patients can be individually assigned to a particular cancer subtype and then treated with appropriate tailored therapies [2]. In addition, early diagnosis is another important element of a successful cancer treatment. This becomes increasingly possible due to the rapid development of modern technology and deeper understanding of cancer biology.

**Figure 1:**
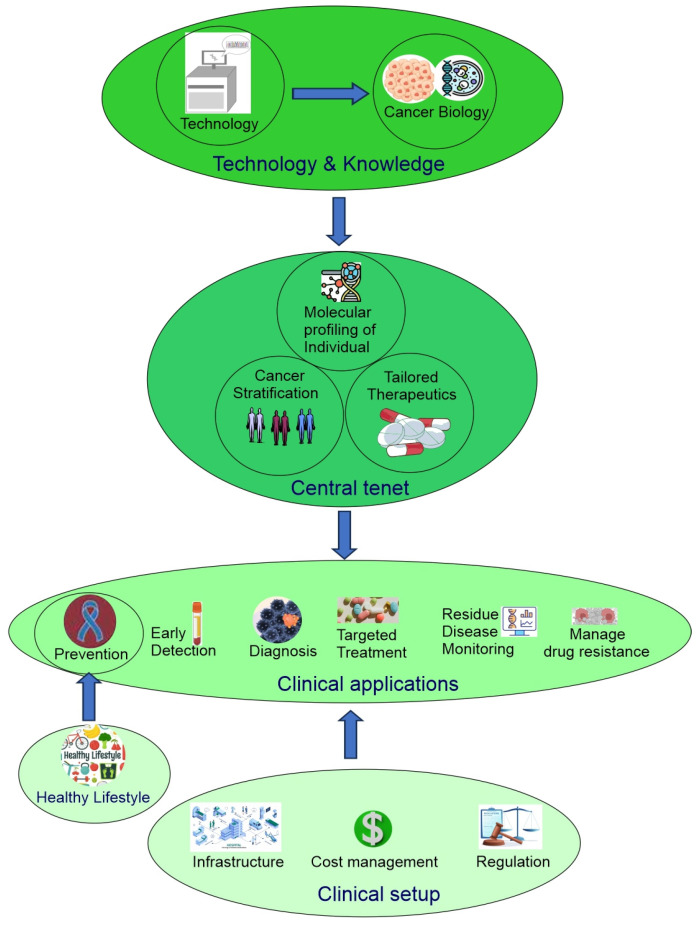
Overview of precision oncology.

Now, precision oncology has expanded to include modern technology (big data, single-cell spatial multiomics, molecular imaging, liquid biopsy, CRISPR gene editing, stem cells, organoids), a deeper understanding of cancer biology (driver cancer genes, single nucleotide polymorphism, cancer initiation, intratumor heterogeneity, tumor microenvironment ecosystem, pan-cancer), cancer stratification (subtyping of traditionally defined cancer types and pan-cancer re-classification based on shared properties across traditionally defined cancer types), clinical applications (cancer prevention, early detection, diagnosis, targeted therapy, minimal residual disease monitoring, managing drug resistance), lifestyle changes (physical activity, smoking cessation, reduced alcohol consumption, sunscreen), cost management, public policy, and more (Figure 1) [2,4].

In this review, we will focus on the various pillars of precision oncology, with an emphasis on emerging trends, challenges, and future perspectives.

## 2. Emerging and Maturation of Technologies in Precision Oncology

**Figure 2:**
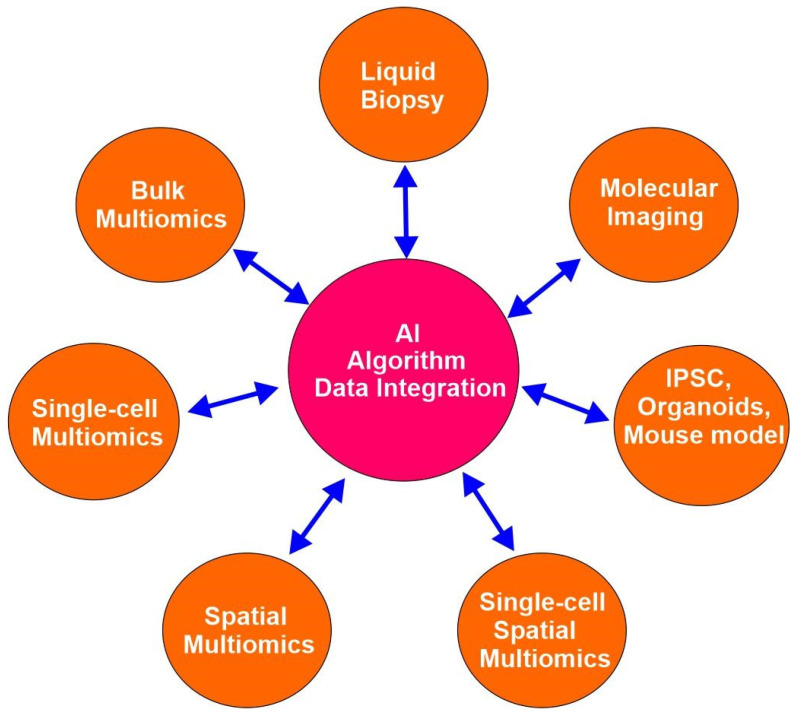
Emerging technologies in precision oncology.

Advanced emerging technologies have revolutionized cancer research since the completion of the human genome project in the early 21st century and empowered precision oncology. These technologies include, but are not limited to bulk multiomics, single-cell multiomics, spatial multiomics, single-cell spatial multiomics, organoids, induced pluripotent stem cells (iPSCs), CRISPR gene editing, liquid biopsy, molecular imaging, and artificial intelligence (AI) (Figure 2). These technologies facilitate the acquisition of highly detailed and multidimensional insights into biological systems.

### 2.1. Single-Cell Multiomics

**Figure 3:**
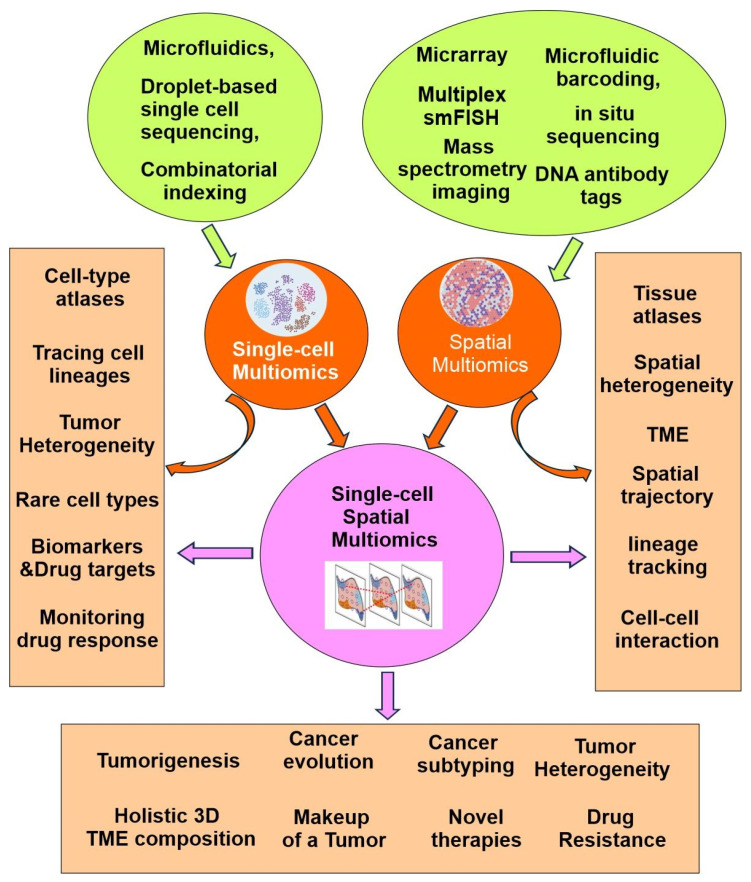
Technical basis and applications of single-cell multiomics, spatial multiomics and single-cell spatial multiomics.

The fundamental high-throughput technology in omics is Next-Generation Sequencing (NGS). NGS sequences millions of DNA fragments at once, producing enormous amounts of molecular data efficiently, quickly, and at a lower cost. It has advanced nearly every area of omics research. However, the initial one-dimensional bulk omics struggle with many issues, such as intratumor heterogeneity and tumor microenvironment (TME). Recently developed technologies, including single-cell multiomics, spatial multiomics, and single-cell spatial multiomics have allowed for a much deeper understanding of the novel aspects of tumor biology (Figure 3).

Single-cell sequencings can reveal the specific effect of an individual cellular component. Since the first report of single-cell genome-wide mRNA sequencing in 2009 [5], various single-cell cell sequencing methods have been developed, including single-cell DNA sequencing for genomics, single-cell DNA methylome sequencing quantifying DNA methylation, single-cell ATAC-seq (Assay for Transposase-Accessible Chromatin using sequencing) investigating chromatin accessibility at the single-cell level, single-cell proteomics quantifying the expressed proteome in an individual cell, and single-cell metabolomics [6,7].

Single-cell omics technologies offer high-resolution analysis of cellular diversity, overcoming the limitations of bulk methods that mask individual cell differences [7]. The emerging trend in single-cell omics is to integrate multimodal omics data within a single-cell to generate a holistic and comprehensive picture of cellular processes. Multimodal omics can help clarify complex cellular interactions, regulatory networks, and molecular mechanisms.

Importantly, the single-cell multiomics approach has revolutionized our ability to dissect cellular mechanisms by allowing for the concurrent measurement of multiple biomolecular layers from the same cell. This integrative perspective is particularly valuable for understanding cellular heterogeneity in complex tissues, disease microenvironments, and developmental processes. As a result, researchers are now able to trace lineage relationships, map cell fate decisions, and identify novel biomarkers with greater precision than ever before (Figure 3) [6,7].

#### 2.1.1. Technological Advancements in Single-Cell Multiomics

Single-cell multiomics techniques allow for the simultaneous analysis of genomics, transcriptomics, epitranscriptomics, epigenomics, proteomics, and metabolomics in individual cells, making them valuable for studying complex cellular processes. A more comprehensive understanding of cellular function and regulation can be achieved by integrating multimodal information [6,7].

Recent advancements in single-cell multiomics have been driven by the development of high-throughput platforms and innovative analytical methods that allow simultaneous measurement of multiple molecular modalities. Technologies such as microfluidics, droplet-based sequencing, and combinatorial indexing have greatly increased the scale and accuracy of single-cell analyses (Figure 3). These innovations have not only expanded the capabilities of researchers to interrogate cellular complexity, but have also facilitated the integration of transcriptomic, genomic, epigenomic, and proteomic data within individual cells, paving the way for more holistic and nuanced biological insights.

##### Single Nuclei RNA-Seq (snRNA-Seq)

While single cell RNA-seq (scRNA-seq) offers valuable insights, it has limitations. Firstly, it requires tissues to be processed into single-cell suspensions, a step involving enzymatic incubation at high temperatures that can cause artifacts and noise, detectable only after sequencing. Additionally, this process may favour easily dissociable cells, leading to biased cellular representation. Single nuclei RNA-seq (snRNA-seq) addresses these issues by profiling gene expression from isolated nuclei, making it suitable for archived or hard-to-dissociate tissues. This method reduces bias in cell type isolation and better reveals the cellular basis of disease, enabling identification of otherwise difficult-to-isolate cell types.

In 2019, Wu et al. conducted a genomic study of the kidney, comparing scRNA-seq and snRNA-seq methods. Their results indicated that snRNA-seq achieves a comparable gene detection rate to scRNA-seq in adult kidney tissue, while also offering advantages such as compatibility with frozen samples and reduced dissociation bias. That same year, Joshi et al. applied snRNA-seq in a human lung biology study and observed that this approach enabled the identification of cell types from both frozen healthy and fibrotic lung tissues without bias [8]. Sequencing adult mammalian heart tissue is challenging due to difficulties in tissue dissociation without cellular damage. Nevertheless, in 2020, researchers in Germany reported sequencing an adult mammalian heart using snRNA-seq and provided data on cell-type distributions within the tissue [9].

#### 2.1.2. Opportunities Provided by Single-Cell Multiomics

With the maturation of the technology and the increase in number of profiled cells, single-cell multiomics has empowered the identification of previously unknown rare cell types, the elucidation of cellular compositions, the characterization of cellular interactions in complex tissues, and the expansion of single-cell atlases for both diseased and healthy human tissues. The Human Cell Atlas (HCA) project seeks to profile all human cells in order to construct a comprehensive reference map. Since its launch, the HCA has assembled single-cell atlases at a large scale. The data collected by the project includes the fluxome, genome, metabolome, proteome, and transcriptome. Overall, the advent of single-cell multiomics has revolutionized the field of precision oncology, providing novel strategies for cancer management (Figure 3).

##### Tracing Cell Lineages

Single-cell multiomics enable the concurrent acquisition of data on genomic copy number variations, DNA methylation, nucleosome occupancy, and transcriptome profiles at the single-cell level. This comprehensive approach facilitates the identification of novel cell types and elucidates their functional roles within specific biological lineages [10,11]. Recent studies show that single-cell lineage analysis helps explain drug resistance in glioblastoma [12] and clarifies which chronic lymphocytic leukemia lineages respond to treatment using combined transcriptome and methylome data [13].

##### Production of Cell-Type Atlases of Various Organs

Single-cell multiomics datasets grow more complex with additional samples, conditions, and acquisition methods. Integration methods aim to reduce batch effects while preserving biological variation. The Cancer Genome Atlas (TCGA) provides multiomics data focused on cancer, including over 20,000 primary cancer samples and their matched normal counterparts from 33 cancer types. This dataset includes genomic, epigenomic, transcriptomic, and proteomic information. TCGA is the largest repository of cancer multiomics data and is widely used in scientific research.

##### Tumor Heterogeneity, Immunology, and Genetics

Single-cell multiomics helps researchers to define the states of tumor and immune cell and the interaction between them, identify predictive biomarkers of treatment response, infer the complex nature of antigen–immune receptor dynamics, and guide the development of therapeutics for multiple cancer types.

Here are some recent findings by using single-cell multiomics technologies. Single-Cell Multiomics revealed prostate cancer heterogeneity [14], uncovered intra-cell-line heterogeneity across human cancer cell lines [15], and revealed differentiation pathways in acute myeloid leukemia [16]. Single-cell multiomics was also employed to characterize the cancer immunosenescence landscape, which shows that patients exhibiting higher levels of immunosenescence signature have poorer prognoses [17]. Single-cell multiomics identified region-specific characteristics of glioblastoma, facilitating complementary therapeutic strategies [18]. Single-cell multiomics identified chronic inflammation as a driver of TP53-mutant leukemic evolution [19], revealed that FABP1 + renal cell carcinoma drive tumor angiogenesis through the PLG-PLAT axis under fatty acid reprogramming [20], revealed context-dependent roles for susceptibility genes by mapping transcriptomes and chromatin accessibility of 117,911 human lung cells from ever- and never-smokers [21], identified key mediators of lasting CAR T therapy response from 695,819 pre-infusion CAR T cells [22].

### 2.2. Spatial-Multiomics

Although single-cell multiomics has provided valuable insights into cellular heterogeneity, it lacks spatial context. Single-cell multiomics methods require cell dissociation, resulting in loss of information about cellular physical interactions. This spatial context is essential to many biological processes. Spatial multiomics overcomes this limitation by enabling the precise localization and molecular characterization of individual cells within their tissue environments [23]. The advancements demonstrated by these innovative techniques are expected to build upon—and potentially surpass—the considerable progress achieved through dissociated single-cell approaches (Figure 3) [6,7,24].

#### 2.2.1. Technological Advancements in Spatial Multiomics

Methods for spatial mono-omics including spatial transcriptomics, epigenomics, proteomics and metabolomics have progressed tremendously in the last decade. Now, the emerging trend is to integrate spatial mono-omics methods to perform spatial multiomics. Spatial multiomics facilitates the concurrent analysis of various data modalities within a single tissue section (Liu, 2024 [23]).

Most spatial multiomics techniques build upon existing mono-omics methods, such as array-based spatial transcriptomics, microfluidic barcoding, DNA antibody tags, multiplex smFISH, in situ sequencing, and mass spectrometry imaging (Figure 3) [24]. These approaches can be applied independently to adjacent tissue sections, sequentially or concurrently on the same section, depending on analyte quality and compatibility. However, integrating multiomics data remains technically challenging, requiring advanced computational and statistical tools. Interpretation is further complicated by environmental variability and technical noise.

#### 2.2.2. Applications of Spatial-Multiomics

Spatial multiomics advances precision oncology by providing detailed insights into tumor cell composition and tissue architecture. It also reveals cell–cell interactions and provides key insights into the spatial organization of the TME. Spatial multiomics is able to identify key cell–cell signaling pathways that drive tumor progression and affect treatment response. As a results, spatial multiomics has broad applications, including spatial-based heterogeneity in cancers, spatial-related crosstalk in tumor immunology, spatial trajectory and lineage tracking of tumor cells, biomarker discovery, disease mechanisms, drug target identification, and the development of novel therapies (Figure 3) [25].

Recently, various spatial multiomics methods have been employed in precision oncology. For example, spatial transcriptomics, metabolomics, and proteomics were integrated to analyze glioblastoma and demonstrated bidirectional tumor-host interdependence [26]. Another study uses digital spatial profiling (DSP) technology to quantitate transcript and protein abundance in spatially distinct regions of metastatic prostate cancer [27]. Another spatial multiomics study reveals the impact of tumor ecosystem heterogeneity on immunotherapy efficacy in patients with advanced non-small cell lung cancer treated with a bispecific antibody [28]. Spatial multiomics has also been used to map immune activity in HPV-negative head and neck squamous cell carcinoma [29]. Through spatial multiomics, it is revealed that SPP1+ fibroblasts play a pivotal role in determining metabolic heterogeneity and promoting metastatic growth of colorectal cancer liver metastasis [30]. A comprehensive spatial multiomics strategy involving imaging mass cytometry (IMC), spatial proteomics, single-nucleus RNA-seq (snRNA-seq) and multiplex immunofluorescence have been developed for profiling breast cancer oligo-recurrent lung metastasis [31]. Spatial multiomics is also used to analyze tumor-stroma boundary cell features to predict breast cancer progression and therapy response [32]. Combined imaging-based spatial metabolomics and lipidomics with microarray-based spatial transcriptomics are employed to visualize intratumor metabolic heterogeneity in gastric cancer [33]. Spatial multiomics has also been used to investigate the spatial distribution of intratumoral microbiota in breast cancer and their interactions with the local TME [34].

### 2.3. Single-Cell-Spatial Multiomics and Human Tumor Atlas Network (HTAN)

As discussed above, both single-cell multiomics and spatial multiomics have its unique advantage and limitations. To maximize the advantages and overcome the limitations, single-cell spatial multiomics have emerged as the most powerful tool to reveal the molecular profiles of both normal and cancer cells/tissues in a temporal-spatial dynamic way. Integrating spatial multiomics data with single-cell multiomics data opens possibilities to add anatomical dimensions to existing datasets and to better understand cell-type-specific molecular profiles in humans (Figure 3).

Single-cell spatial multiomics were initially developed as single-cell spatial transcriptomics by combining single-cell RNA-seq with spatial transcriptomics. With the inclusion of more and more other single-cell mono-omics and spatial mono-omics, it truly becomes single-cell spatial multiomics. However, currently, based on the purpose of the study, most studies only combine select single-cell mono-omics (such as single-cell transcriptomic, single-cell proteomics, single-cell epigenomics, etc.) with select spatial mono omics (such as spatial transcriptomics, spatial proteomics, and spatial epigenomics, etc.).

Single-cell spatial multiomics-based international initiatives have emerged to facilitate the advancement and application of precision oncology. The Human Tumor Atlas Network (HTAN) is a prominent representative. HTAN facilitates scientific collaboration to unify samples, analytical techniques, and resources into comprehensive atlases illustrating tumor evolution. By broadening the spatial perspective on molecular, cellular, and tissue characteristics, HTAN provides a multidimensional insight into cancer biology that significantly supports progress in precision oncology.

Since 2018, HTAN researchers have gathered single-cell spatial multiomics data and used advanced analytical methods to better understand tumor ecosystems across various organs and types. This project demonstrates how spatial and single-cell data advances knowledge of cancer progression and supports the discovery of new tumorigenesis mechanisms. Following are some important contributions from the HTAN researchers, as well as from the broad scientific community (Figure 3).

#### 2.3.1. Tumor Evolution and Microenvironment Interactions in 2D and 3D Space

Using spatially resolved single-cell genomics, transcriptomics, and proteomics, a study characterized 131 tumor spatial transcriptomics sections in six cancer types. The research described aspects of spatial tumor evolution via interactions with the local microenvironment in two- and three-dimensional space, offering information relevant to tumor biology [35].

#### 2.3.2. Temporal Recording of Development and Precancer

Lineage tracing methods based on CRISPR evolving barcodes are utilized in studies of mouse development and mouse models of colorectal cancer. The findings reveal the polyclonal make-up of early cancers and their decreasing clonal diversity during the transition to advanced cancers. This result was also observed in human colorectal cancer samples at various stages of progression [36].

#### 2.3.3. Molecular Pathways Associated with Early Tumorigenesis in Familial Adenomatous Polyposis (FAP)

Single-cell spatial multiomics, including transcriptomic, proteomic, metabolomic and lipidomic, were integrated to profile 93 samples, consisting of normal mucosa, benign polyps, or dysplastic polyps, from six persons with FAP. The results generated by this research reveal key genomic, molecular, and cellular events during the earliest steps in colorectal cancer formation and potential mechanisms of pharmaceutical prophylaxis [37].

Besides HTAN associated studies, single-cell spatial multiomics has been employed by the scientific community to study various aspects of many cancer types.

#### 2.3.4. Cancer Subtype Stratification

Single-cell spatial multiomics has been instrumental in cancer subtype stratification. The spatial immunophenotypes were assigned in TNBC by integrating spatial phenotypes and immunity effectors with multiplexed immunofluorescent imaging, scRNA-seq and TCR repertoire analysis, which helped to elucidate T cell evasion pathways in response to ICB [38]. By integrating data generated with scRNA-seq and spatial transcriptomics sequencing, as well as bulk RNA sequencing, proteomic analysis, and genome sequencing, a recent study reveals novel subtypes of hepatocellular carcinoma [39].

#### 2.3.5. Cancer-Associated Fibroblasts (CAF)

Through integrative analyses of over 14 million cells from 10 cancer types across 7 spatial transcriptomics and proteomics platforms, a recent study validates and characterizes four distinct spatial CAF subtypes, which facilitates novel approaches to target and modulate CAFs [40]. Integrating spatial and single-cell transcriptomes reveals the role of COL1A2(+) MMP1(+/−) cancer-associated fibroblasts in ER-positive breast cancer [41].

#### 2.3.6. Tumor Heterogeneity and Holistic TME Cellular Components

While tumor heterogeneity and TME cellular components have been studied by single-cell omics and spatial omics. Single-cell spatial omics allows for a deeper and broader understanding.

An integrated single-cell spatial multiomics landscape of WHO grade 2–4 diffuse gliomas has revealed locoregional metabolomic regulators of glioma growth [42]. A high-throughput single-nucleus snRNA-seq and snATAC-seq multiomics dataset from matching “core” and “margin” dissections in four distinct grade 4 High-Grade Gliomas (HGG) patients are combined with new spatial transcriptomics data from two additional HGG samples to evaluate “core-to-margin” transition, which provides insights into the residual disease biology of tumors and the microenvironment at the infiltrative margin [43]. By integrating 12 spatial and single-cell technologies, a recent study characterized tumor neighborhoods and cellular interactions in three skin cancer types [44]. In neuroblastoma, single-cell multiomics from a mouse spontaneous tumor model and spatial transcriptomics from human patient samples are used to dissect the transcriptional and epigenetic landscapes governing developmental states and demonstrates tumor developmental plasticity [45].

Multimodal single-cell-resolved spatial proteomics has been employed to reveal pancreatic tumor heterogeneity [46]. Single-cell spatial multiomics has been used to analyze the immune dysfunction in pancreatic and revealed novel mechanisms underlying disease development [47].

### 2.4. Patient-Derived Tumor Organoids (PDTO)

The identification of features unique to individual patients and tumors is important in advancing precision medicine and conducting preclinical studies in cancer treatment. Cancer is characterized by substantial inter- and intra-tumor heterogeneity, resulting in varying clinical responses to standard treatments among patients. This heterogeneity has hindered precision cancer treatments until the emergence of PDTOs. PDTOs reliably mimic primary tumor structures, functions, molecular traits, and genomic changes, while also allowing for genomic and environmental manipulation. PDTOs are an indispensable tool in precision oncology to mimic illnesses, explore mechanisms, identify innovative therapeutic targets, screen and assess novel drugs in a high-throughput manner, and customize treatment regimens for individual cancer patients [48,49,50,51,52].

#### 2.4.1. Technological Advancements in PDTO

An organoid is a three-dimensional structure created in vitro that replicates key functional, structural, and biological characteristics of an organ in a simplified and smaller form. Organoids are derived from either pluripotent or tissue-resident stem (embryonic or adult) or progenitor or differentiated cells from healthy or diseased tissues, such as tumor s [53].

Researchers have used a range of three-dimensional (3D) cell culture methods to replicate tissue and organ complexity. The spheroid model was introduced in the early 1970s [54]. These compact spherical structures, typically over 1 mm in size, are mainly derived from immortalized cell lines. In 1987, the optimization of cell culture conditions enabled mammary epithelial cells to assemble into 3D spheroids and ducts [55]. In 1998, human pluripotent stem cells (hPSCs) were successfully extracted from human blastocysts, opening the door for the development of regenerative medicine [56]. In the early years of the 21st century, iPSCs were successfully generated through the reprogramming of mouse and human fibroblasts, which has greatly advanced the development of three-dimensional organoid models [57].

Over the past decade, PDTOs have contributed to developments in 3D culture in precision oncology research. In 2009, it was shown that an adult intestinal stem cell expressing the LGR5 receptor, isolated from mice, could be cultured to form structures and cellular diversity resembling the crypts and villi of the intestinal epithelium [58]. Organotypic cultures have since been created from various primary tumors and have shown to better mimic original tumor features than traditional cell lines. An effective PDTO can grow, store, and freeze cells that maintain the genetic and histological features of the original tumor [51].

#### 2.4.2. Application of PDTOs

PDTOs can model TME cell diversity and interactions by co-culturing with non-cancerous cells, such as CAFs and different immune cells. As a result, PDTOs demonstrate utility in various aspects of cancer research, including cancer biology, cancer therapies, disease progression and tumor niche factor requirements [51].

##### Cancer Biology

Cancer initiation

Organoids including PDTOs are increasingly being used to understand cancer biology. Organoids have been used to model the stages of tumorigenesis in various types of tumors. The formation of organoid from cells can be evaluated to reveal different stages of tumor evolution. Observing this process shows that inactivating tumor suppressor genes (such as TP53, PTEN, or APC) and activating oncogenes (such as KRAS) are keys to tumor formation. Studying the shift from healthy to tumor organoids helps clarify the molecular basis of tumor initiation and may reveal novel biomarkers for early cancer diagnosis, advancing precision oncology [51]. Tumor organoid models may also be relevant for mimicking the genomic evolution of tumors, as shown by a recent study in bladder cancer [59].

Mechanism of drug resistance

Organoids can mimic clinical tumor responses, making it possible to reliably track resistance development and identify underlying mechanisms. Furthermore, combined with imaging techniques, the different responses of cells within the PDTO can be analyzed separately to overcome the challenges imposed by cell heterogeneity.

Recently, researchers have used molecular comparisons between PDTOs from chemotherapy-treated patients and those from untreated tumors to find targetable signaling pathways [60]. Another method is growing tumor organoids from chemotherapy-treated PDXs in mice to measure parameters not assessable in vivo [61]. Organoids have also been used to model acquired resistance in pancreatic cancer [62].

Tumor Heterogeneity and TME

In recent years, a three-dimensional (3D) organoid culture of human tumor tissue has gained recognition as a cost-effective and representative platform for modelling cancer heterogeneity and tumor microenvironment interactions in vitro.

Studies have modeled patient-to-patient heterogeneity by creating ‘living biobanks’ of organoids derived from cancer tissues. For example, pancreatic cancer organoids from a genetically and phenotypically comprehensive cohort of 138 patient tumor samples have been established, which revealed population-level genetic and transcriptomic signatures associated with anticancer drug responses [63]. A biobank consisting of 55 colorectal cancer organoid lines was established, representing a range of tumor phenotypes, including both primary and metastatic lesions [64].

PDTO has been used for studying TME heterogeneity, such as non-cancerous cell roles, niche-specific signalling factors, and changes in extracellular matrix composition. Recent studies have aimed to create culture platforms that better reflect TME cell diversity and their interactions. It is shown that native CAFs and immune cell types could be retained in PDTOs to test personalized immunotherapies [65]. Schmalzier et al. also introduced a platform to evaluate cancer immunotherapies using human CAR-engineered natural killer cells targeted at patient-derived CRC organoids [66].

##### Clinical Application

PDTOs have diverse uses: they enable high-throughput anti-cancer drug screening, assess toxicity and side effects, help identify therapeutic targets and candidates, predict drug sensitivity, support personalized clinical therapies, model diseases, and advance research with CRISPR-Cas9 gene editing [49].

A major clinical application of PDTOs is drug screening. The PDTO model allows for high-throughput screening of therapeutic options, making it possible to identify tumor subtypes that could preferentially benefit patients. Tumor organoids derived from rectal cancer patients have been used to conduct drug sensitivity tests. These findings were used to guide patient treatment with a success rate of 88% in terms of effectiveness [67]. Another study utilized lung cancer organoids for high-throughput drug screening and prediction of drug response, which could potentially facilitate personalized cancer treatment strategies [68]. Through high-throughput drug screening, a study evaluated 76 drugs across 30 PDTOs obtained from pancreatic tumors and showed the potential of the PRMT5 inhibitor EZP015556 in inhibiting MTAP-negative tumors [69]. Another organoid platform was developed for high-throughput screening of 2427 drugs to test their sensitivity in colorectal cancer [70]. In gastric cancer, nine PDTOs were exposed to 37 clinical and developmental compounds, confirming their responsiveness to existing targeted therapies [71]. Similarly, colorectal cancer-derived PDTOs were screened with 83 molecules, highlighting links between drug efficacy and specific genetic alterations [51].

PDTO panels are utilized to identify predictive molecular signatures—including genomic, transcriptomic, and proteomic markers—associated with treatment responses. Increasing evidence demonstrates that PDTOs can accurately forecast the responsiveness of their source tumors to anticancer therapies [72].

#### 2.4.3. Challenges and Limitations

Despite the rapid development, wide application, and unique ability to address difficult questions facing precision oncology, current PDTOs have several limitations and challenges. The experimental protocols are still too complicated, which hinders the timely delivery of reliable data to the clinicians for optimal clinical management. Organoid creation and maintenance are expensive, raising concerns about cost. Tumor tissues are complex and varied, but current organoids do not fully reflect this diversity in vitro. Success rates for generating cancer organoids differ widely across tumor types. Despite improvements in protocols, techniques remain variable and difficult to reproduce due to non-standardized tissue sources, processing methods, media, and matrices [48,49,51].

Moving forward, the emerging trend is to develop more complex models that recapitulate in vivo structure and function as faithfully as possible and enhance its application value in clinical treatment and drug development. Tumor organoids can be co-cultured with non-tumor cells or iPSC-derived organoids, which enables the investigation TME and the effects of immune cells in the absence of an immune system. Moreover, organoids can be integrated with advanced technologies such as 3D printing and organ chips to create engineered organs.

### 2.5. Liquid Biopsy

**Figure 4:**
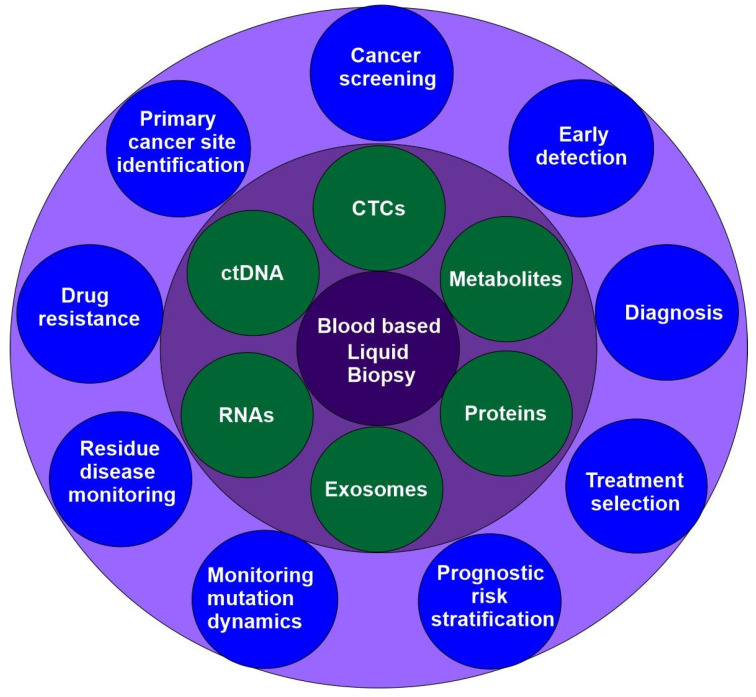
Blood-based liquid biopsy: methods and applications in precision oncology.

Liquid biopsy is an emerging precision medicine method that examines blood and other body fluids for insights into a patient’s health. Liquid biopsy is a non-invasive method used to identify and analyze biomarkers, including circulating tumor cells (CTCs), cell-free DNA (cfDNA), circulating carcinoma proteins, circulating miRNAs, and exosomes. This approach enables real-time monitoring of cancer mutations and a patient’s genomic profile. Compared to traditional tissue biopsies, liquid biopsies offer increased speed, safety, and practicality, and make it possible to collect serial samples for observing tumor evolution over time (Figure 4) [73,74]. Liquid biopsy could revolutionise medical diagnosis, but faces challenges like few tumor -derived components, low specificity, and limited progress in isolating biomarkers.

Liquid biopsies include both blood-based and non-blood-based methods. Due to its prominence, blood-based biopsies also are referred to as liquid biopsies. Non-blood-based liquid biopsies include urine analysis, breath analysis, and saliva analysis, which were recently reviewed by other articles [74,75,76,77] and will not be discussed here.

#### 2.5.1. Circulating Tumors Cells (CTCs)

CTCs, first identified by Thomas Ashworth in 1869, are cancer cells released into the bloodstream from tumor sites [74,78]. In recent decades, they have been detected in various cancers, including prostate, ovarian, gastric, colorectal, bladder, renal, lung, glioblastoma, and melanoma [79,80]. CTCs are traditionally characterized as nucleated cells present in a blood sample that exhibits negative staining for the ubiquitous leukocyte marker CD45 while demonstrating positive staining for epithelial cytokeratin.

Analysis of CTCs through liquid biopsy has been used for decades in various cancer types, showing applications in early diagnosis, prognostic risk assessment, disease monitoring, and informing personalized treatment decisions. CTCs are intact tumor cells, which enables CTCs to serve as a source of information at multiple molecular levels, including DNA, RNA, protein, and metabolites. In addition, CTC monitoring uses routine blood draws rather than tissue biopsies, offering a less invasive and more repeatable method for detecting cancer and assessing its progression.

CTCs are highly heterogeneous, have a brief half-life of 1–2.4 h, and exist in very low concentrations in peripheral blood [74]. Although millions of CTCs enter the bloodstream daily from each gram of tumor tissue, they make up only a tiny proportion of blood cells—sometimes as few as one per 10 billion [80]. Facing these challenges, the clinical potential of CTC-based diagnosis has not been fully realized due to the limitation of the existing methods and devices to harvest CTCs and their clusters from peripheral blood [81]. Therefor, the advancement of CTCs in precision oncology is significantly dependent on the advancement of CTC enrichment technology.

All CTC isolation strategies exploit unique properties of CTCs including distinct immunological, molecular, and/or bio-physical properties. These strategies fall into two categories: those that result in up to 10^4^-fold CTC enrichment and those that achieve up to 10^8^-fold enrichment [80,82]. Approaches for 10^4^-fold enrichment include direct visualization of CTCs, capture based on cell size or physical characteristics, positive immunoselection, and hematopoietic cell depletion for negative enrichment. For 10^8^-fold enrichment, further purification is necessary and typically involves labeling residual white blood cells and CTCs with fluorescence-conjugated antibodies, identifying CTCs optically, and sorting them individually. The use of droplet-based scRNA-seq technologies is another approach [80,82].

In addition to CTCs, metastasis is facilitated by dissemination of cell clusters containing CTCs. Some CTCs travel in clusters, ranging from doublets to dozens of cancer cells tethered together. Some clusters may include tumor-derived fibroblasts, blood-derived leukocytes, or other cells. Other methods have also been developed to isolate CTC clusters. For example, negative enrichment of CTC clusters from peripheral blood samples based on the apoptosis resistance of malignant cells of tumorigenic origin and enrichment by immunocytochemistry profiling [81,83].

#### 2.5.2. Circulating Tumor DNA (ctDNA)

ctDNA comprises small fragments of DNA that are released by tumor cells into the blood and tissue fluids. Plasma ctDNA shares molecular traits with tumor tissue and can be measured easily from blood, making it a potential biomarker for primary cancer screening. Many studies demonstrate that the plasma ctDNA concentration is associated with tumor location, size, and extent of disease [84]. For example, it is shown that ctDNA positivity is in 42%, 67% and 88% of patients with stage I, II, and III NSCLC, respectively [85]. Advances in DNA technology have increased interest in detecting ctDNA through liquid biopsy.

Half a century ago, scientists realized that plasma levels of free DNA increased in cancer patients [86]. Specific mutated genes began to be identified in the blood of cancer patients in the form of ctDNA about 20 years ago, including KRAS and PIK3CA in patients with colorectal cancer [87], EGFR in breast cancer patients [88] and in NSCLC patients [88,89]. In 2014, the European Medicines Agency (EMA) approved the use of ctDNA for detecting EGFR mutations in NSCLC [90]. ctDNA assay was recommended for cancer patients by the Society for Medical Oncology (ESMO) Precision Medicine Working Group in 2022 [91].

Different cancers display distinct epigenetic patterns and genomic breakpoints in ctDNA, which help predict primary tumor types. While these markers might not be exclusive to one subtype, comparing DNA methylation, nucleosome footprints, and genetic alterations in ctDNA to reference databases can still identify cancer type [84,92]. Multiple platforms have been on the market including ExoDx prostate Intelliscore and SelectMDx for prostate cancer [93], Shield [94], Freenome test, and Epi proColon [95] for colorectal cancer, Viome CancerDetect for oral and oropharyngeal cancer [96], and a test for breast cancer [97].

Because ctDNA is highly fragmented and scarce, increasing detection sensitivity remains a key research focus. Currently, ctDNA detection is well-developed and includes two main types: targeted PCR-based methods and nontargeted next-generation sequencing (NGS) techniques.

ctDNA now is heavily explored for multicancer early detection (MCED) with several developed platforms [98,99,100,101,102]. The sensitivity of these tests varies between 29% and 98%, while the specificity is close to 100% [84]. In recent years, more comprehensive studies have been conducted to screen and diagnose more cancers with or without symptoms. In 2023, the first large-scale prospective evaluation of an MCED diagnostic test in a symptomatic population was conducted in England and Wales (SYMPLIFY) [103]. The NHS-Galleri trial (ISRCTN91431511) was designed to evaluate whether an MCED test that screens asymptomatic individuals for cancer can lower the incidence of late-stage cancer [104]. Concurrently, a large multi-institutional prospective study in the United States, PATHFINDER, examined the feasibility of cancer screening using MCED testing. This approach utilized next-generation sequencing of cell-free DNA from peripheral blood and focused on conducting diagnostic investigations for participants who tested positive for a cancer signal. The study detects cancer-specific methylation patterns from ctDNA in more than 50 distinct cancer type [105]. This study supports the feasibility of MCED screening for cancer and underscores the need for further research investigating the test’s clinical utility.

#### 2.5.3. Exosomes

Exosomes offer a promising option and can enhance liquid biopsy diagnostics in some cases. Exosomes carry less information than CTCs, but much more information than ctDNAs as they contain DNAs, RNAs, proteins, lipids, and others. Exosomes are secreted by living cells, offering a more representative view of the cellular state than cell-free DNA (ctDNA), which is primarily released by dying cells. The lipid bilayer of exosomes provides a stable environment for the biomolecules they carry, protecting them from degradation and making them reliable diagnostic indicators. The rich chemistry of exosomes offers various therapeutically relevant diagnostic options. Exosomes have potential applications in cancer screening and early diagnosis due to the information they provide regarding viable tumor cells. A cancer cell can release about 20,000 vesicles in 48 h. Tumor-related exosomal biomarkers have been studied in cancers such as lung, breast, kidney, prostate, and colorectal cancer [106].

In order to be used as a biomarker, exosomes must first be separated from the respective biofluid. Many methods have been developed over the years including the conventional methods such Centrifugation-Based Methods, Ultrafiltration Methods, Precipitation Methods, Field Flow Fractionation Methods, Chromatographic Methods, and Affinity Binding-Based methods [107], as well as newly developed microfluidic technology-based methods. Advances in microfluidic technology enable efficient and portable exosome separation and detection, paving the way for point-of-care applications [107,108].

Combining different exosomal components (RNA, proteins, lipids) and integrating them with other liquid biopsy markers (like ctDNA) can improve diagnostic sensitivity and specificity. Mutations in exosomal RNA can complement ctDNA signals, enhancing mutation detection sensitivity. Studies indicate that cfDNA combined with exosomal RNA outperforms cfDNA analysis alone [109].

#### 2.5.4. Other Biomarkers

Liquid biopsies can also analyze other tumor-derived material like proteins, metabolites, and RNAs.

### 2.6. Non-Invasive Imaging Methods

Conventional oncology image analysis, utilizing modalities such as ultrasound, X-ray, CT, and MRI, has traditionally depended on manually defined features for the interpretation and evaluation of clinical images. The low precision has impeded the accurate diagnosis of the cancer, leading to more unnecessary biopsies. Recent breakthroughs in oncologic imaging leads to the emerging and development of cancer molecular imaging, which have transformed cancer diagnosis, treatment planning, and monitoring.

#### 2.6.1. Cancer Molecular Imaging

Cancer molecular imaging refers to the non-invasive visualization of molecular and cellular processes that are associated with neoplasia, including proliferation, glucose metabolism, and receptor expression. Various molecular imaging modalities are used, such as magnetic resonance, optical, and nuclear imaging [110]. Cancer molecular imaging primarily relies on dual-modality techniques, including PET/CT (introduced commercially in 2001) and SPECT/CT (commercialized in 2004), which integrate anatomical, metabolic, and functional data to enhance diagnostic accuracy [111]. Other molecular imaging technologies have also improved significantly, enhancing their capabilities to support precision medicine. For example, MRI is able to generate high-resolution anatomic images of soft tissue. Optical imaging is empowered by the use of bioluminescence, fluorescence, and near-infrared imaging. Ultrasound imaging offers real-time, portable, nonradioactive options [77,111].

Recent advances in molecular imaging include super-resolution fluorescence microscopy for nanoscale cell visualization, DNA-PAINT achieving sub-20 nm resolution, and photoacoustic techniques for deep tissue imaging. Advances in machine learning and imaging data have led to the identification of new biomarkers and quantitative features in cancer molecular images [111].

The RECIST system (Response Evaluation Criteria in Solid Tumors) is a standardized set of rules used in oncology to objectively assess how solid tumors in patients respond to cancer treatment by measuring changes in lesion size on standard imaging. However, it can be slow and sometimes inaccurate when assessing targeted therapies. Molecular imaging techniques have the potential to overcome this weakness due to its ability to quantitatively assess the response at the cellular, subcellular, or even molecular level [112].

As precision medicine continues to evolve, the application of molecular imaging will continue to evolve alongside it, encompassing a wider range of clinical and research settings.

#### 2.6.2. Omics Imaging, Radiomics and Imaging Genomics

Due to the widespread availability of omics data and improvements in imaging technologies, integrating biomedical image information with omics data has become feasible. This integration process can reveal the links between the micro-level molecular information generated by various omics with the macro-level structural and functional information provided by biomedical images. This newly emerging interdisciplinary field is named omics imaging.

Bridging imaging and omics factors and exploring their connections has the potential to provide important new insights into the phenotypic characteristics and molecular mechanisms of cancer development. These, in turn, will impact the development of new diagnostic, prognostic, therapeutic, and preventive approaches, becoming an important component of precision oncology [113].

Radiogenomics uses big data to aid cancer diagnosis and treatment decisions, offering insights into tumor biology and identifying key imaging biomarkers. These approaches have been validated in a variety of tumors including glioblastoma, breast cancer, liver cancer, colorectal cancer, gastric cancer, lung cancer, ovarian cancer, prostate cancer, retinoblastoma, head and neck squamous cell cancer [114].

#### 2.6.3. Whole Slide Imaging (WSI)

Whole Slide Images (WSIs) are high-resolution digital scans of tissue slides that are revolutionizing precision medicine by enabling AI-driven analysis for more accurate tumor detection, treatment response prediction, and biomarker discovery. These large digital files offer pathologists and researchers enhanced accessibility, quantitative insights, and the ability to integrate with machine learning for personalized patient care and drug development [115,116]. Recently, a whole-slide pathology foundation model, Prov-GigaPath, was established and pretrained on 1.3 billion 256 × 256 pathology image tiles in 171,189 whole slides from Providence, a large US health network comprising 28 cancer centres. The achieves state-of-the-art performance on various digital pathology tasks, demonstrating the importance of real-world data and whole-slide modelling [117].

### 2.7. AI Powered Data Integration, Machine Learning and Deep Learning

The primary objective of precision medicine is to combine substantial amounts of data from various databases into analytic frameworks that support the creation of diagnostic and therapeutic methods that are individualized and context specific. This data is generated by various layers of high-throughput technologies such as multiomics. However, it faces challenges over its dimensionality, interpretability, predictability, and high computational power demand. Integration of AI into precision oncology provides the best solution to overcome these challenges. AI can be used to build analytical models of complex disease to improve diagnostic accuracy, optimizing treatment strategies, and enhancing patient care through personalized interventions and remote monitoring that predict personalized health conditions and outcomes. AI has rapidly emerged as a transformative force in precision oncology and has revolutionized various aspects of cancer care from diagnosis to treatment [118,119].

#### 2.7.1. Principles and Workflow

A workflow for data integration by AI modeling in precision oncology include the following steps: (A) Selection of the data sources. A wide variety of data sources with diverse features are available for selection. (B) Data collection and pre-processing. Different approaches to data collection and pre-processing are needed to deal with the diverse data sources. (C) Integration of the diverse and heterogenous data through data processing and modeling. (D) Application of the integrated data to precision medicine for diagnosis, treatment strategies, and outcome prediction [118].

#### 2.7.2. Subtypes of AI in Medicine

AI in medicine manifests in various subtypes, each with unique functionalities.

##### Machine Learning

Machine learning is a branch of AI that uses algorithms to mimic human learning. It helps uncover patterns in medical data that may escape experts. Common healthcare algorithms include decision trees, support vector machines, and random forests, which analyse large datasets to predict treatment protocols and outcomes [119,120].

##### Deep Learning

Deep learning constitutes a branch of machine learning that leverages multilayered neural networks to accomplish tasks including classification, regression, and representation learning. The discipline draws conceptual frameworks from biological neuroscience and focuses on organizing artificial neurons into hierarchical layers, which are “trained” to process information efficiently. Deep learning techniques are particularly effective for analyzing intricate data patterns, such as those found in images, natural language, and genomic sequences. Some common deep learning network architectures include fully connected networks, deep belief networks, recurrent neural networks, convolutional neural networks (CNNs). CNN is a powerful deep learning method that enables the precise detection of malignant lesions.

##### Transfer Learning

Transfer learning is a technique in machine learning in which knowledge learned from a task is re-used in order to boost performance on a related task.

##### Natural Language Processing

Natural language processing enables machines to understand and generate human language. It is used to extract information from medical records, summarize patient histories, generate tailored treatment plans, transcribe patient-doctor interactions, and produce medical reports. Much patients’ information is stored in free-text format in electronic health records… Natural language processing techniques can be used to extract information from unstructured clinical notes and social media data, enabling analysis of patient experiences and disease trends [121].

##### Computer Vision

Computer vision enables machines to analyse images and videos, playing a crucial role in tasks like medical imaging, identifying issues in X-rays or MRIs, and supporting surgeries.

#### 2.7.3. Application in Precision Oncology

##### Cancer Detection

AI has been applied for the detection of almost all types of cancers. AI-based tools for detecting breast cancer via mammography represent a rapidly advancing field, with many systems progressing toward real-world clinical application [122]. Several AI products have received FDA clearance for assisting radiologists with the detection of breast cancer from mammograms, as well as in interpreting MRI and breast ultrasound examinations. A recent clinical study in Sweden demonstrated that integrating AI into mammography screening workflows significantly decreased radiologists’ workload—by approximately 44%—without compromising diagnostic performance [123]. The application of AI to predict future breast cancer risk is also explored by several studies and these AI risk prediction algorithms have been shown to outperform traditional risk models [124,125,126].

Similar studies have applied deep learning to colonoscopy images and video for colorectal cancer screening and reported increased detection rate [127,128,129]. AI has also been used to localize lung nodules for purpose to predict lung cancer risk [130,131] and to predict prostate cancer risk [132,133]. Advanced deep learning models have been developed for skin-cancer detection [134].

##### Cancer Treatment

AI algorithms are also being developed to improve treatment, including designing personalized treatments and monitoring treatment efficacy. For example, AI is used to predict the response to neoadjuvant chemotherapy in TNBC [135] and in predicting the prognosis for breast cancer [136] and colorectal cancer [137].

##### Cancer Biology

AI has been used for the interpretation of germline and somatic mutations observed in cancer. For example, deep learning was employed in a recent study to detect pathogenic germline variant in prostate cancer and melanoma [138]. The recently developed deep learning-based models include Dig [139], AlphaMissense [140] and CancerVar [141].

Multiple AI methods have developed cell-of-origin prediction, which is particularly relevant for cancers of an unknown primary [142,143,144,145]. The OncoNPC model, trained using sequencing data from tens of thousands of tumor samples spanning over 20 cancer types, has demonstrated strong predictive power [143]. Another study analyzed transcriptomics data from 37 cancer types provided by TCGA to identify cancer tissue-of-origin specific gene expression signatures [144]. Cross-protein transfer learning substantially improved disease variant prediction in a recent study [145]. 

Various AI algorithms have also been established for detecting cancer specific neoantigens [146,147] and associated T-cell receptors [148,149]. Deep learning on pathology images are also used to analyze spatial organization and molecular correlation of tumor-infiltrating lymphocytes to better understand TME [150]. Various AI models have also been developed for the purpose of cancer subclassification. For example, a comprehensive deep learning and transfer learning analytic model has been introduced for skin cancer classification [151].

## 3. Complete Understanding of the Tumor Biology

A complete understanding of tumor biology is the key for cancer prevention, early detection and diagnosis, and effective treatment, which is also the ultimate goal of precision oncology. With the rapid development of novel and powerful technologies as discussed above, we now have a much deeper understanding of cancer biology. The discovery of novel knowledge and the emergence of novel concepts and principles have significantly advanced precision oncology.

### 3.1. Tumorigenesis/Cancer Initiation

Tumorigenesis is a complex, multistep process in which oncogenic mutations within normal cells initiate clonal expansion. This progression is significantly modulated by additional factors, including environmental tumor risk elements and epigenetic modifications, both of which can substantially affect early clonal proliferation and malignant transformation independently of mutational events [152]. A deeper understanding of the earliest molecular events holds promise for translational applications, predicting individuals at high-risk of tumor and developing strategies to intercept malignant transformation.

#### 3.1.1. Genomics and Cancer Genes

Mutation processes that commence during embryological development contribute to clonal evolution by introducing variability within the tumor cell population. Certain mutations arise in cancer driver genes, promoting positive selection [4,152]. Driver genes are a specific subset of genes that harbor mutations directly contributing to the initiation and progression of cancer. These genes often regulate critical cellular processes such as the cell cycle, apoptosis, and DNA repair. Mutations in driver genes give a growth advantage to the cells and are thus positively selected; they promote clonal expansion and can lead to uncontrolled proliferation [4,152,153].

Early genomic alterations encompass point mutations, short insertions, deletions, structural variations, copy number changes, gene fusions, and methylation differences. Analysis of exome data from 33 tumor types has identified 229 genes undergoing positive selection [154]. Further investigation into somatic mutations across more than 28,000 tumors representing 66 cancer types has revealed 568 cancer-associated genes and provided insights into their roles in tumorigenesis [155]. Utilizing data from 2658 cancers across 38 tumor types provided by the Pan-Cancer Analysis of Whole Genomes (PCAWG) Consortium under the International Cancer Genome Consortium (ICGC), sixteen distinct signatures of structural variation have been characterized [156]. In a recent study, MethSig was developed to specifically identify candidate DNA methylation driver genes of cancer progression and relapse from 22 cancer types. Chromosomal instability (CIN) leads to extensive losses, gains, and rearrangements of DNA. The resulting genomic complexity is recognized as a hallmark of cancer; however, no comprehensive framework currently exists to systematically quantify various forms of CIN or assess their impact on clinical phenotypes across cancer types. This study analyses the extent, diversity, and origin of chromosomal instability in 7880 tumors spanning 33 distinct cancer entities. Seventeen copy number signatures defining specific CIN types have been identified, which facilitate drug response prediction and support the discovery of novel therapeutic targets [157]. Increasing evidence indicates that clones exhibiting aberrant epigenetic reprogramming display heightened tumor susceptibility within morphologically normal tissues [158]. For instance, during the precancerous progression of lung cancer, the epigenome advances through distinct stages, ultimately resulting in substantial intra-tumor heterogeneity in invasive lesions. Phylogenetic analyses based on methylation abnormalities closely mirror those derived from somatic mutations, implying concurrent methylation and genetic evolutionary processes [159].

#### 3.1.2. Clonal Expansion

Through intensive studies, the process of cancer initiation begins with healthy tissue through clonal expansion [4]. Single-cell spatial multiomics and many other cutting-edge technologies have been employed to study cancer clonal expansion from normal tissue in many different cancer types [160,161,162]. However, many issues remain unsolved and have become emerging research areas. For example, how can cancer driver events exist in normal tissues? Some studies suggest that selection pressure is different under different environments. *NFKBIZ* mutations may offer benefits in chronic inflammation but are often selected against in cancer, limiting tumor development [83]. The impact of this competition on early tumorigenesis is unclear. Recent research shows most new oesophageal tumor s are removed through competition with mutant clones in nearby normal tissue [163].

Understanding mechanisms driving the clonal expansion of normal tissue toward early cancer initiation will provide insight into cancer prevention and treatment and is thus an important topic being explored.

#### 3.1.3. Environmental Carcinogenesis

The causal links between environmental exposures and cancer initiation has been gradually established [164,165,166,167]. These environmental factors include chemical and radical insults, unhealthy metabolic behaviors such as alcohol consumption and smoking, and specific pathogenic infections. These environmental factors induce genetic and epigenetic alterations in transformed cells and have profound impacts on microenvironmental components that predispose to tumor initiation. For example, KRAS G12C mutations in NSCLC are generated through smoking-related mutagenesis [168,169].

However, many environmental factors cause cancer by mechanisms other than mutations [170]. This non-mutagenic carcinogenesis is an emerging hot research topic [4]. One likely mechanism is the deregulation of cancer-related gene expression through epigenetic modifications [4,171,172,173]. Another mechanism may be through chronic inflammation. Some studies indicate that many environmental factors cause local inflammation, which provides a different selection pressure in favor of cancer initiation [152,173]. The mechanisms by which inflammation facilitate early tumorigenesis include oxidative stress, DNA damage, mutation clone expansion, cell proliferation, and cell survival [19,83,174,175].

### 3.2. Tumor Heterogeneity

Thanks to the development of single-cell spatial multiomics, the concept of tumor heterogeneity has moved onto the center stage of cancer research. Both intertumoral and intratumoral heterogeneity are crucial factors for the complete understanding of molecular foundation of tumors. While intertumoral heterogeneity has been well studied and largely reflected by molecular stratifications based on driver genes, pathways, and expression profiles, intratumor heterogeneity (ITH) is much less understood and is an emerging area being extensively studied [2,4].

ITH commonly occurs in malignant tumors due to changes in genetic, epigenetic, transcriptomic, proteomic, metabolic, and microenvironmental factors. This complexity can drive tumor progression, treatment resistance, and impact clinical diagnosis, prognosis, and treatment strategies. While multiomics technologies now provide comprehensive mapping of ITH at multiple molecular levels, there are ongoing challenges with applying these findings clinically.

ITH makes most targeted drugs less effective, as tumor cells often have varied clonal or subclonal genetic profiles.

In recent years, advanced technologies like the single-cell omics, spatial omics and CRISPR-based lineage tracing have substantially enriched our understanding of ITH. Each technology offers distinct advantage. Single-cell multiomics allow the dissection of ITH at cellular resolution. The single-cell layer of resolution provides the most precise lens through which the clonal complexity, lineage dynamics, and evolutionary trajectories of tumors can be observed [176]. A recent study integrated bulk genomics data with co-occurrences of mutations from single-cell RNA sequencing data to reconstruct clonal trees in high-grade serous ovarian cancer and breast cancer and achieved high resolution and high-fidelity results [177]. Spatial omics such as spatial transcriptomics holds great promise in deciphering the complex heterogeneity of cancer by providing localization-indexed gene expression information [178]. Epigenomics is able to uncover the regulatory landscape of tumor heterogeneity. Proteomics provides functional diversity in cancer cells. However, due to the limitation of each technology, the integrated analysis of single-cell spatial multiomics become the most adopted method to explore ITH. Some recent studies highlight this progress in various tumor types [14,179,180].

Due to their heterogeneity, the bulk tumor might include a diverse collection of cells harbouring distinct molecular signatures with different levels of sensitivity to treatment. This heterogeneity might be across different disease sites (spatial heterogeneity) or change over time (temporal heterogeneity). Heterogeneity provides the fuel for resistance. Indeed, drug resistance has been linked to the heterogeneity of many cancers [181,182]. Using single-cell transcriptomics, a recent study of pediatric Burkitt lymphoma reveals intra-tumor heterogeneity and markers of therapy resistance [183]. An accurate assessment of tumor heterogeneity is essential for the development of effective therapies. Current strategies to combat heterogeneity-related drug resistance include (1) upfront treatment with potent pan-inhibitory TKIs, instead of reserving them for the second-line; (2) design of dosing schedules based on the mathematical model of the tumor heterogeneity; and (3) combinatorial approaches that pair therapies targeting the predominant, drug-sensitive population of clones in addition to the various subsets of drug-resistant and drug-tolerant cells [181]. More recently, a study showed that intratumor heterogeneity of EGFR expression mediates targeted therapy resistance and formation of drug tolerant microenvironment. Pharmacological induction of EGFR with epigenetic inhibitors increases the sensitivity of resistant cells to EGFR inhibition. This indicates that intrinsic drug resistance may be addressed through combination therapies [184].

### 3.3. Holistic TME Ecosystem

A tumor consists of various cell types that interact within the tumor microenvironment (TME). The TME includes cancer cells, stromal cells, immune cells, and signaling molecules, forming a complex cellular ecosystem. In the absence of single-cell spatial omics, it is difficult to either distinguish tumor-associated signals of other components or understand the spatial distribution and interaction among cells in the tumor and TME. The advancement of single-cell spatial multiomics has propelled a profound paradigm shift in the understanding of tumor and TME cellular components and heterogeneity. Recent studies with single-cell spatial multiomics have revealed multiple tumor-enriched cell types including immune cells, CAFs, and other stromal cells with diverse functions.

However, the current understanding of the makeup of a tumor is still incomplete and coarse-grained. More research is needed to achieve a complete depiction of the tumor. We need to determine if we have identified most cell types or much more needs to be discovered, if all cell types are heterogeneous with multiple subtypes, what are the functions of these identified cells, what cell types are universal and what are cancer type-specific, and what are the critical factors leading to the makeup of the TME?

A recent study analyzed over 14 million cells from 10 cancer types using seven spatial omics platforms, identifying four conserved CAF subtypes with distinct spatial patterns, cell interactions, and transcriptomic profiles. These subtypes influence tumor microenvironment features like immune cell infiltration and patient survival, offering new directions for targeting CAFs and advancing cancer research [185]. Another study with single-cell/single-nucleus RNA sequencing and spatial transcriptomics on 62 samples from 25 pancreatic ductal adenocarcinoma (PDAC) patients have uncovered distinct cellular subtypes involved in neural invasion in pancreatic cancer [186]. Single-cell spatial multiomics suggests SPP1+ fibroblasts may drive metabolic variation and support colorectal cancer liver metastasis [30]. Phenotypic diversity and plasticity in CRC are key factors in tumor growth, spread, and therapy resistance. Single-cell spatial multiomics data are generated from metastatic colorectal samples, which reveals regenerative and inflammatory cancer cell states, identifies AP-1 and NF-dB as key regulators of the regenerative cell states, and locates the regenerative cells at the invasive edge in an immunosuppressive niche [187].

Immune evasion is a major obstacle in cancer treatment, enabling tumors to avoid immune detection and complicating therapies, which leads to poorer patient outcomes [188]. Further research should identify key cell types and suppression mechanisms involved in tumor immune evasion and clarify whether these processes are universal or specific to cancer types. The tumor microenvironment influences immune response through multiple pathways, collectively enabling tumors to avoid immune detection. Currently, several mechanisms have been identified. TME may promote immune evasion by recruiting and polarizing immune cells into immunosuppressive phenotypes by secreting cytokines that can either stimulate or suppress immune evasion, by influencing the expression of immune checkpoint molecules, and by metabolic reprogramming [188].

A recent study using single-cell transcriptomic data from around 200 human colorectal donors identified distinct TME subtypes and described how cancer cells use varied immune evasion mechanisms [189]. It is also shown recently that spatial architecture of myeloid and T Cells orchestrates immune evasion in lung cancer [190]. Another research classified CRC into five TME subtypes with distinct response rates to immunotherapy [191].

## 4. Cancer Stratification

As an essential objective of precision oncology, cancer stratification is the task of classifying a cancer into distinct patient subgroups based on specific patient characteristics, which can then guide treatment decisions based on which subgroup a patient belongs to [192]. An active and growing body of work is exploring different approaches for identifying homogeneous patient subgroups, ranging from qualitative models that are based on clinical observations alone to quantitative models that integrate measurements from diverse high-throughput biotechnologies [193,194]. Cancer stratification can benefit both cancer research and the cancer treatment.

### 4.1. Brief History

For centuries, cancer was thought to be a single disease. Hippocrates, in 400 B.C., described it as one condition that persisted despite surgery. It has since been realized that cancer is not a single disease; instead, it is a collection of hundreds of different diseases. Although cancer stratification has been conducted for many years, it was conducted as a by-product of clinical experience in the old time. When clinicians noticed the presence of patterns or groups of outlier patients, they performed a more thorough (retrospective or prospective) study to confirm their existence. However, this type of subtyping was limited by the expertise and resources of individual doctors.

Collective efforts have been made to stratify cancers systematically based on their location (tissue, organ, and system), morphology, and histology. Cancers are traditionally classified four ways: (I) broadly, by tissue, organ, and system; then by (II) specific type, and (III) grade according to WHO classifications; and (IV) finally by spread according to the Tumor Node Metastasis (TNM) system. These classifications have played a crucial role in shaping clinical oncology, guiding cancer research, and informing the education of oncologists and pathologists [195].

Starting in 2000, the WHO classifications began to include biologic and molecular–genetic features. The completion of the Human Genome Program and the advancement of high-throughput biotechnologies have provided the means for measuring differences among individuals at the cellular and molecular levels. The cost of measuring various “–omics” data (such as genomic, proteomic, and metabolomics data) has decreased significantly, enabling scientists to collect such data on a large number of patients. Thus, research has shifted toward computationally driven approaches to identify subtypes. These developments led to the era of precision oncology, which have had a strong impact on cancer diagnosis and treatment [4,195].

**Figure 5:**
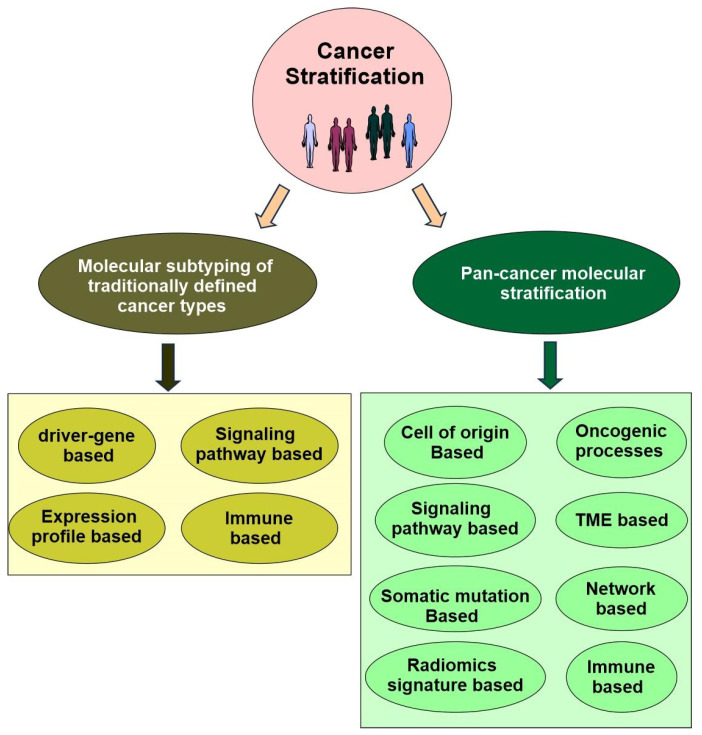
The landscape of current and emerging molecular stratification of cancers in precision oncology.

In precision oncology, molecular cancer stratification now falls into two categories. One is the molecular subtyping of traditionally defined cancer types, which is the initial strategy and is still advancing now. The other one is pan-cancer molecular stratification, which is more newly developed and has the potential to revolutionize precision oncology (Figure 5).

### 4.2. Molecular Subtyping of Traditionally Defined Cancer Types

While there are many ways to further subtype traditionally defined cancer types, the major strategies include cancer driver gene based-stratification, signaling pathway alteration-based stratification, expression profile-based stratification, and immune based stratification (Figure 5).

#### 4.2.1. Cancer Driver Gene-Based Stratification

Traditional molecular cancer stratification relies on genetic and epigenetic drivers and expression profiles. This method identifies anti-tumor targets and guides personalized cancer therapy development. For example, breast cancer caused by *erbB2* driver gene is classified as a HER2-enriched breast cancer subgroup. This stratification allows the development of tailored therapy to specifically target HER2. The development and clinical application of the therapeutic monoclonal anti-HER2 antibody is one the first successful examples. Another successful example is the classification of the non-small cell lung cancer (NLCSC) caused by the mutation of driver gene *erbB1*. Based on this stratification, a small molecular inhibitor gefitinib was developed and successfully applied for treating this group of patients. A third example is the stratification of cancers caused by BRAF V600E mutations. V600E is a driver mutation in many tumors, including melanoma. The targeted therapeutic drug Vemurafenib was developed, which is effective in treating melanoma harboring V600E mutation [196]

Identifying molecular cancer drivers is critical for precision oncology. More comprehensive research has been done in recent years to identify novel cancer driver genes and explore the opportunities to target these cancer driver genes specifically in multiple types of cancers. A recent analysis of 9423 tumor exomes from 33 cancer types in The Cancer Genome Atlas projects (TCGA) identified 299 driver genes, which are associated with specific anatomical locations and cancer or cell types [154].

A study analysed genomic data from 20,331 tumor s across 41 cancer types, cataloguing driver mutations in 727 cancer genes. Mutation rates vary by cancer type, with high involvement of tumor suppressor genes (94%), oncogenes (93%), transcription factors (72%), kinases (64%), cell surface receptors (63%), and phosphatases (22%). The analysis also showed that cancer gene mutations commonly co-occur rather than occur exclusively across all cancer types. Furthermore, the study reported that patients with tumor s exhibiting different combinations of gene mutation patterns demonstrate varying survival outcomes. These results offer further information about the genetic features of cancer and contribute to our understanding of the mechanisms underlying various forms of cancer [197].

A study of whole-genome sequencing data from 10,478 patients with 35 types of cancer, part of the UK 100,000 Genomes Project, identified 330 candidate driver genes—74 of which were previously unknown in cancer. About 55% of patients had mutations linked to treatment response or resistance and clinical trial eligibility. Computational analysis revealed new targets for compounds that could be considered for upcoming trials. This work constitutes one of the most comprehensive initiatives to date to identify cancer driver genes in a real-world setting and evaluate their significance in guiding precision oncology [198].

#### 4.2.2. Signaling Pathway Alteration-Based Stratification

It is quite often that cancer is not caused by a single gene modification, but multiple alterations along a signaling cascade. The alteration of an oncogenic signaling pathway is the combined effects of multiple alterations; cancers that share these common features could be classified into a specific group and treated with targeted therapeutic drugs.

Signaling pathways have been used to further subtype specific cancers for many years. For example, a large volume of research has explored oncogenic signaling pathways associated with TNBC, including the cell cycle, DNA damage response, and androgen receptor (AR) signaling pathways, to identify more efficient targeted therapies [199]. In addition, the dysregulation of Wnt signaling in TNBCs has also been explored for its potential biological roles in molecular subtyping [101].

Pathway-based clustering methods use biological pathway databases [200]. Pathifier identified pathways significantly linked to survival in glioblastoma and colorectal cancer [201]. Several pathways identified by Pathifier were significantly associated with survival of glioblastoma patients. R-Path Cluster identified two subtypes of glioblastoma and several pathways associated with the cancer progression [202]. As a follow up, a pathway-based deep clustering method (PACL) for molecular subtyping of cancer was introduced. The patient groups clustered by PACL may correspond to subtypes which are significantly associated with distinct survival distributions [202].

In addition, pan-cancer analysis of signaling pathways have contributed significantly to pan-cancer molecular stratification [203], which will be discussed later.

#### 4.2.3. Expression Profile-Based Stratification

In precision oncology, the molecular stratification of cancer is mostly based on the molecular profiling of the cancers. Initially, the molecular profiling is mostly based on the genomics of the cancer, which then expanded to the expression profiles defined the transcriptomics and proteomics. More recently, epigenomics, metabolomics, microbiomics and/or metagenomics were all included in the molecular profiling of patients. Advances in omics stem from high-throughput technologies, which have transformed medical research since the human genome project was completed. These technologies now provide detailed snapshots of biological systems at extremely high resolution.

The molecular stratification based on multiomics is mostly to further subtype cancers defined by histology or anatomic origin. For breast cancer, TNBC was further subtyped to 4 or more subgroups based on Omics. In 2011, Lehmann et al. from VICC used k-means clustering method to study gene expression profiles of 587 TNBCs and separate them into six subtypes [204]. In 2016, it was determined that transcripts in the IM and MSL subtypes did not originate from cancer cells, leading to a refined TNBC classification into four types: BL1, BL2, LAR, and M. In accordance with the VICC subtyping system, Burstein et al. applied mRNA profiling to 198 TNBC samples using non-negative matrix factorization (NMF) clustering and identified four TNBC subtypes with distinct molecular characteristics in 2015 [205]. Subsequently, researchers at Fudan University Shanghai Cancer Center (FUSCC) sequenced 465 TNBC samples and used multiomics data to define four mRNA-based clusters, further proposing targeted treatment strategies for each cluster [206]. The FUSCC group profiled the polar metabolome and lipidome in 330 TNBC samples and 149 normal breast tissues, creating a metabolomic atlas. They identified three TNBC subgroups: C1 (enriched ceramides and fatty acids), C2 (upregulated metabolites related to oxidation and glycosyl transfer), and C3 (least metabolic dysregulation) [207].

Colorectal cancer (CRC) has been characterised by four consensus molecular subtypes (CMSs), identified through extensive gene expression profiling, with each subtype linked to distinct oncogenic mechanisms [208]. CMS classification for CRC includesCMS1 (14%)—hypermutated, microsatellite unstable, strong immune activation; CMS2 (37%)—epithelial, WNT/MYC signaling; CMS3 (13%)—epithelial, metabolic dysregulation; and CMS4 (23%)—mesenchymal, TGF-β activation, stromal invasion, angiogenesis. Mixed samples (13%) may indicate transitional or heterogeneous phenotypes. CMS is currently the most robust and interpretable CRC classification, forming the basis for future stratified and targeted therapies [208].

Furthermore, epigenetic profiling has been investigated for tumor stratification and the identification of tumor subtypes. Notably, methylation profiling of cfDNA obtained from blood samples of cancer patients provides valuable insights into tumor subtypes. For example, a recent study on small cell lung cancer developed a cfDNA methylation-based method capable of distinguishing clinically relevant subtypes [209]. This liquid biopsy approach may enhance the clinical application of genomics-informed molecular tumor subtyping.

### 4.3. Pan-Cancer Molecular Stratification

While molecular subtyping has been mostly employed to further subtype the traditional cancer types organized by histology or anatomic origin, a new trend is to develop pan-cancer classification. Cancers in different organs and tissues have similarities at the molecular level, and their similarity is even greater than that of the same tumors, suggesting the possibility for the re-classification of cancers by pan-cancer molecular stratification across the traditionally defined cancer types. Pan-cancer molecular stratification allows more accurate and effective targeted treatment of cancer patients, which becomes an important part of precision oncology.

The current major strategies for pan-cancer molecular stratification include cell of origin-based stratification, oncogenic processes-based stratification, oncogenic signaling pathway-based stratification, TME-based stratification, somatic mutation-based stratification, network-based stratification, radiomics signature-based stratification, and immune-based stratification (Figure 5).

#### 4.3.1. Pan-Cancer Molecular Stratification Based on the Cell of Origin

The Cancer Genome Atlas (TCGA), backed by NCI and NHGRI, advanced pan-cancer molecular research by creating the Pan-Cancer Atlas. This resource is based on data from over 11,000 tumors across 33 cancer types, offering a comprehensive view of tumor origins and development in humans. This data generated the first wave of pan-cancer molecular stratification.

The cell of origin in cancer is defined as the normal cell that acquires the first cancer-promoting mutation(s) [210]. An analysis of 11,000 samples from 33 cancer types shows that cell-of-origin is key to tumor classification [211]. Researchers proposed a new 28-cluster system for classifying human tumors. Most molecular subtypes are heterogeneous, with the largest cluster including 25 tumor types. The findings from this study indicates that cell-of-origin influences, but does not fully determine, tumor classification. Traditional tumor classification by histology or site should be complemented with the Pan-Cancer Atlas molecular taxonomy, which groups tumor s by shared molecular features across different tissues [211].

Indeed, a study analysis of molecular data on 2579 tumors from TCGA of invasive breast cancer and four gynecological cancers including uterine corpus endometrial carcinoma, high-grade serous ovarian cystadenocarcinoma, cervical squamous cell carcinoma and endocervical adenocarcinoma, and uterine carcinosarcoma, identified shared and unique molecular features, clinically significant subtypes, and potential therapeutic targets. This study utilized 16 key molecular features to identify five prognostic subtypes and constructed a decision tree that assigns patients to these subtypes using six features which can be analyzed in clinical laboratories [212].

Another study analyzed 921 adenocarcinomas of the esophagus, stomach, colon, and rectum to examine shared and distinguishing molecular characteristics of gastrointestinal tract adenocarcinomas (GIACs). Five molecular subtypes Epstein–Barr virus (EBV)-positive, hypermutated-single-nucleotide variant predominant (HM-SNV), microsatellite instability (MSI), chromosomal instability (CIN), and genomically stable (GS) are identified among GIACs [213]. Squamous cell carcinomas can also be classified into different subgroups based on genomic, pathway network, and immunologic features [214]. Renal cell carcinoma (RCC) encompasses multiple histologically distinct malignancies, each characterized by unique genetic drivers, clinical trajectories, and therapeutic responses. Analysis of 843 RCC cases spanning the three principal histologic subtypes—including 488 clear cell RCCs, 274 papillary RCCs, and 81 chromophobe RCCs—demonstrates distinct characteristics for each subtype. These findings provide a foundation for the development of targeted therapeutic and management strategies tailored to individual RCC subtypes [215].

More recently, it has been revealed that the cell-of-origin influences pancreatic cancer subtypes, which provide insight into the fundamental impact that the very earliest events in carcinogenesis can have on cancer evolution [216]. Diffuse large B-cell lymphoma (DLBCL) is a heterogeneous group of cancers classified together on the basis of morphology, immunophenotype, genetic alterations, and clinical behavior. DLBCL is one of the earliest cancers being subtyped based on the cell of origin. Through the years, the cell of origin subtypes of DLBCL have been refined by various methods [206,217,218]. Recently, a refined subtyping based on integrated multiomic analysis on 228 relapsed/refractory DLBCL samples was able to distinguish subtype-specific mechanisms of treatment resistance and relapse, which facilitates the development of personalized treatment of this high-risk group [219].

By applying five different machine learning approaches to multiomic data from 8791 TCGA tumor samples comprising 106 subtypes from 26 different cancer cohorts, a very recent study developed models based upon small numbers of features, which can classify new samples into previously defined TCGA molecular subtypes. These models are further validated by using external datasets [220].

It is worth noting that cell-of origin has also been used to further subtype specific cancers. For example, diffuse large B-cell lymphoma (DLBCLs) is grouped into three distinct molecular subtypes based on the putative cell of origin (COO): the activated B-cell-like (ABC), the germinal B-cell-like (GCB), and the unclassifiable subtype as defined by array-based gene expression profiling [221,222]. Further studies have determined cell-of-origin subtypes of diffuse large B-cell lymphoma using gene expression in formalin-fixed paraffin-embedded [217] and have developed a platform independent protein-based cell-of-origin subtyping of diffuse large B-cell lymphoma in formalin-fixed paraffin-embedded tissue [223].

#### 4.3.2. Pan-Cancer Molecular Stratification Based on the Oncogenic Processes

The Pan-Cancer Atlas provides a panoramic view of the oncogenic processes that contribute to human cancer. A comprehensive study which analyzed the oncogenic processes across 11,000 tumors from 33 cancer types revealed that germline genome affects somatic genomic landscape in a pathway-dependent fashion; genome mutations impact expression, signaling, and multiomic profiles; and mutation burdens and drivers influence immune-cell composition in the microenvironment [224]. The results offer the opportunity for pan-cancer molecular stratification and targeted therapies based on these important oncogenic processes and will provide a foundation for future personalized cancer care.

A companion study with the TCGA performed immunogenomics analyses of more than 10,000 tumors and have identified six immune subtypes that encompass multiple cancer types. These six immune subtypes differ by somatic aberrations, microenvironment, and survival, which defines immune response patterns impacting prognosis. These analyses serve as a resource for exploring immunogenicity across cancer types and set a foundation for future targeted studies to further advance precision oncology [225]. A recent study processes 364 individual tumors cross cancer types by clustering upon 10 features and identifies 12 unique tumor archetypes spanning cancer type. Each archetype concentrates similarities in additional immune and tumor features. Dominant archetypes aid in tumor classification and identifying therapeutic targets [226].

We discuss above that cancer driver genes play an important role in the further subtyping of the traditionally defined cancer types. Recent studies indicate that the analysis of cancer driver genes across multiple cancer types could also serve for pan-cancer molecular stratification. A Pan-Cancer and PanSoftware analysis spanning 9423 tumor exomes from 33 cancer types used 26 computational tools to catalog driver genes and mutations and thus identify 299 driver genes. It reveals that driver genes and mutations are shared across anatomical origins and cell types and thus could form the basis for pan-cancer molecular stratification and targeted treatment [154]. Similarly, another research highlights the driver gene fusion in pan-cancer molecular grouping and its clinical implication [227].

Pan-cancer analysis has also focused on identifying the molecular subtypes associated with specific driver genes. It is shown that SF3B4 is strongly expressed in patients cross cancer types and the expression level is correlated with their survival [228]. By using hepatocellular carcinoma (HCC) as a model, this study illustrates the role of SF3B4 as an oncogenic factor in HCC, highlighting its potential as a pan-cancer therapeutic target and diagnostic biomarker [228]. Analysis of 56 MET-F–positive tumors from an institutional cohort of 91,119 patients (79,864 DNA sequencing plus 11,255 RNA sequencing) uncovered two forms of MET-F pathobiology. MET fusions are primary drivers of tumor growth in multiple tumor types—lung cancer and gliomas—and can be effectively targeted with either type I (crizotinib, capmatinib, tepotinib, and savolitinib) or type II (cabozantinib) MET TKIs, with best responses in tumors harboring fusions with partner homodimerization [229].

In addition, pan-cancer analysis of TCGA data across 11,000 tumors from 33 cancer types have been performed for other important oncogenic process including mRNA splicing [227,230], IncRNA [231,232], enhancer expression [233], and aneuploidy [234].

A recent study presents a novel method based on patient-specific gene regulatory network to identify cancer subtypes based on patient-specific molecular systems. By applying the data collected in TCGA, this study indicates that the novel method is able to identify more clinically meaningful cancer subtypes than the existing subtypes and found that the identified subtypes comprised different molecular features [235]. A recent study introduces a framework for cancer molecular subtyping by identifying specific co-expression modules, generating network features through adjusted edge correlations, and training a deep neural network for multi-class classification. Applied to breast cancer and stomach adenocarcinoma, this method outperforms existing approaches [236].

#### 4.3.3. Pan-Cancer Molecular Stratification Based on Oncogenic Signaling Pathways

In its comprehensive analysis of tumor signaling pathways, the Pan-Cancer Atlas reveals patterns of vulnerabilities that will aid in molecular stratification and the development of personalized treatments and new combination therapies. Using mutations, copy-number changes, mRNA expression, gene fusions and DNA methylation in 9125 tumors cross 33 cancer types, a comprehensive study analyzed ten oncogenic signaling pathways including PI-3-Kinase/Akt, Hippo, Myc, RTK-RAS, Notch, Nrf2, TGFβ signaling, p53, β-catenin/Wnt and cell cycle. It showed that 89% of tumors had at least one driver alteration in these pathways, and 57% of tumors had at least one alteration potentially targetable by currently available drugs. 30% of tumors had multiple targetable alterations, indicating opportunities for combination therapy. Based on this data, tumors can be stratified into 64 subtypes [203].

Seven major metabolic processes and their clinical relevance are analyzed by using molecular data of 9125 patient samples from TCGA. This study provides a pan-cancer classification of metabolic expression subtypes in 33 TCGA cancer types. It shows that metabolic expression subtypes share consistent prognostic patterns across cancer types. Moreover, the metabolic expression subtypes reveal therapeutic targets and are associated with sensitivity to drugs in clinical use [237]. An extensive molecular characterization of 929 ubiquitin-related genes and 95 deubiquitinase genes is performed with multidimensional omics data of 9125 tumor samples across 33 cancer types from TCGA. It reveals consistent prognostic patterns of tumor subtypes defined by ubiquitin pathway genes. This study highlights the importance of the ubiquitin pathway in cancer development and molecular stratification, which lays a foundation for developing relevant therapeutic strategies [238].

Many other signaling pathways including TGF-β [239], Hippo [231], Ras [199], RNA splicing [240], and DNA damage repair [241] are also comprehensively characterized based on TCGA data of 33 cancer types. These analyses establish initial pan-cancer molecular stratification based on oncogenic signaling pathways.

A recent study analyzed proteomic data from 2404 samples and transcriptomic data from 7752 samples across 13 cancers. By comparing normal and tumor tissues, researchers found multiple dysregulated pathways, such as mRNA splicing, interferon, fatty acid metabolism, and complement coagulation cascade. Pan-cancer subtypes were identified by tracking proteins consistently up- or down-regulated across tumor stages. In addition, prognostic risk stratification models are also established based on dysregulated genes. This study reveals that small molecule inhibitors targeting various signaling pathway might be effective treatments for pan-cancer, thereby supporting drug repurposing [242].

Another recent study developed the PathClustNet algorithm, a pathway-based clustering method designed to identify cancer subtypes. This method first detects gene clusters and identifies overrepresented pathways associated with them. Based on the pathway enrichment scores, it reveals cancer subtypes by clustering analysis.

#### 4.3.4. Pan-Cancer Stratification Based on the Tumor Microenvironment

Cancer molecular typing extends beyond cancer cells to include the tumor microenvironment (TME), which significantly impacts clinical outcomes and therapy response. Tumor-infiltrating immune cells influence tumor progression and treatment success, while cancer-associated fibroblasts and stromal cell angiogenic signals also affect outcomes [243,244,245,246,247]. Subtyping cancer by tumor-immune microenvironment profile can enhance personalized treatment.

A comprehensive study developed an accessible transcriptomic analysis platform for TME classification. Analysis of over 10,000 cancer patients revealed four TME subtypes shared by 20 cancers. These subtypes predict immunotherapy response, with immune-favorable TMEs linked to better outcomes. Therefore, TME subtypes serve as broad immunotherapy biomarkers across cancer types, reflecting both tumor and microenvironment features [248].

Cancer-associated fibroblasts (CAFs) are a multifaceted cell population that significantly contribute to reshaping the tumor microenvironment (TME). Through multiple pathways, activated CAFs can promote tumor growth, angiogenesis, invasion, and metastasis, along with extracellular matrix (ECM) remodeling and even chemoresistance. The mutual effects of CAFs and the tumor immune microenvironment (TIME) have also been identified as key factors in promoting tumor progression [249].

A recent study utilized pan-cancer single-cell and spatial transcriptomics analysis to identify the subpopulation of CAFs via senescence related genes, classifying the neuroblastoma patients into high and low risk groups according to median risk score. The low-risk group had a superior survival outcome, an abundant immune infiltration, a different mutation landscape, and an enhanced sensitivity to immunotherapy [250].

Using a training cohort of 88 HCC scRNA-seq samples and a validation cohort of 94 samples, encompassing over 1.2 million cells, a study characterized CAFs in hepatocellular carcinoma patients into three fibroblast subpopulations. Among them, VEGFA + CAFs subtype was induced by hypoxic TME and associated with poorer prognosis [40].

Using single-cell RNA sequencing and spatial transcriptomics, another study investigated the characteristics and functional information of CAF subtypes and explored the intercellular communication between CAFs and malignant epithelial cells in gastric cancer. Cells were classified into nine categories, and analysis showed a correlation between the proportions of epithelial cells and fibroblasts. Six distinct fibroblast subpopulations were also identified, each associated with specific biological processes and immune functions [251].

Through integrative analyses of over 14 million cells from 10 cancer types across 7 spatial transcriptomics and proteomics platforms, a recent study discovered, validated, and characterized four distinct spatial CAF subtypes. These subtypes are conserved across cancer types and independent of spatial omics platforms [40].

#### 4.3.5. Other Approaches for Pan-Cancer Molecular Stratification

Molecular mechanisms underlying cancer metastasis span diverse tissues of origin. A recent study attempted to perform a pan-cancer stratification of cancer metastasis based on cell lineage. The transcriptomes of patient-derived xenografts and patient tumor metastases were collected from 38 studies with over 3000 patients and 4000 tumors. The analysis revealed four expression-based subtypes of metastasis transcending tumor lineage, which underpins metastases beyond tissue-oriented domains and has important therapeutic implications [252].

Network-based stratification (NBS) approaches have been used in a recent study for integrated stratification of three types of cancers including ovarian, bladder, and uterine cancer. It showed that integrated NBS subtypes are more significantly associated with overall survival [253].

Immune response has also been explored for pan-cancer stratification. Many tissue functions are achieved through the complex coordination of immune cells [254]. The immune response is the result of a coordinated action of a collection of immune cell subsets, which allows it to be used as a signature to group tumors to various subtypes [255,256,257]. In a recent study, cell type compositional and transcriptomic data from 364 fresh surgical specimens across 12 tumor types were used to identify conserved tumor immune archetypes. This study successfully identified and validated 12 unique tumor immune archetypes. This data has become a valuable resource for studying cancer immunity and cancer targets, which will greatly improve response to cancer immunotherapy [226].

Pan-cancer molecular stratification based on somatic point mutations have also been explored. Statistical analysis of mutation profiles is challenging due to the low frequency of most mutations, the varying mutation rates across tumor s, and the presence of a majority of passenger events that hide the contribution of driver events. An innovative study provides a method, NetNorM, to represent whole-exome somatic mutation data in a form that enhances cancer-relevant information using a gene network as background knowledge. Using data from 8 cancer types from The Cancer Genome Atlas (TCGA), it is shown that this method improves survival prediction and unsupervised patient stratification [258]. More recently, a new machine learning pipeline has been developed to identify protein-coding genes mutated in many samples to identify cancer subtypes. Data from 12,270 samples collected from the international cancer genome consortium, covering 19 cancer types are tested and 17 different cancer subtypes have been identified [259].

Cancer cells become immortalized through telomere maintenance mechanisms, such as telomerase reverse transcriptase (TERT) activation. A systematic analysis of TERT high and low cancers using multidimensional data from TCGA depicts a telomerase-associated molecular landscape in cancers. Random forest classifiers were generated to identify cancer subtypes [260].

A new framework identifies robust co-expression modules for cancer subtypes, yielding better classification results for breast cancer (BRCA) and stomach adenocarcinoma (STAD) than current methods [236].

Advances in machine learning and deep learning have enhanced cancer diagnosis efficiency. Deep learning radiomics signatures are promising for identifying cancer subtypes, aiding precision treatment [261].

## 5. Targeted Cancer Therapeutics

The importance of tumor stratification is to allow targeted treatment for each subtype. Therefore, an effective therapeutic treatment tailored to each cancer subtype must be available to make precision medicine successful [2]. While targeted therapy for cancer treatment started long before the arrival of the precision medicine era, the rapid expansion and progress of targeted therapy only occurred after the sequencing of the human genome and the development of advanced technology for high-through put analysis of multiomics for tissue samples and single-cells.

### 5.1. Brief History

Before the emergence of targeted therapy, cancers were treated by a combination of surgery, chemotherapy, and radiotherapy.

The origin of radiotherapy dates back to the late XIX century, when it was used for the treatment of breast cancer and epithelioma of the mouth [262]. Modern radiotherapy was established in 1920 when Claudius Regaud demonstrated that radiation fractionation could effectively treat several human cancers by minimizing treatment-related side effects. Despite these advances, surgical intervention remains the sole therapeutic option for advanced or non-solid tumors. A significant milestone was marked in the mid-20th century with the introduction of chemotherapy, following the serendipitous discovery of the first DNA alkylating agent, nitrogen mustard. Mechlorethamine was the initial nitrogen mustard employed clinically as an alkylating agent, primarily used in patients with prostate cancer and various lymphoid malignancies, including Hodgkin’s disease, lympho-reticulosarcomatosis, and lymphatic leukemia [263]. Afterwards, many types of chemotherapy drugs with different action modes were introduced in cancer treatment, leading to significant improvements in survival rate, especially for patients with onco-hematological diseases. Chemotherapy in combination with radiation therapy and surgery has been the dominant treatment for all types of cancers for more than half a century.

However, chemotherapy’s toxicity to normal tissues and the development of drug resistance mechanisms by tumor cells represented important obstacles to overcome [264]. Subsequently, with the understanding of cancer biology including its genetic basis and the discovery of oncogene and tumor suppressor, the idea to target altered proteins to treat cancer emerged and were explored in the final quarter of the last century [2,265]. These approaches have significantly increased the effectiveness of treatments and the survival rates of cancer patients.

The real development of targeted therapy comes at the beginning of the 21st century with the success of the human genome project and the rapid development of molecular biology technology, which provides the precise detection of genome, transcriptome, and proteome changes. By using these methods, researchers can explore the mechanisms underlying cancer and design new targets specific for the pathogenic molecules [266]. The new regimen delivers personalised, efficient treatment by targeting abnormal genes or proteins. Recent advances in gene editing and cell therapy have rapidly advanced targeted therapies.

To date, besides the development of novel targeted drugs, pharmaco-omics is employed to identify drugs best for each individual. Pharmaco-omics selects the right drugs and dosages for each individual based on the person’s particular genetic/molecular makeup, as well as a person’s environment, diet, age, lifestyle, and state of health. Moreover, functional precision medicine is applied to examine the direct response of patient-derived cancer cells to various selected drugs in vitro.

### 5.2. The Development and Current Status of Targeted Therapies

**Figure 6:**
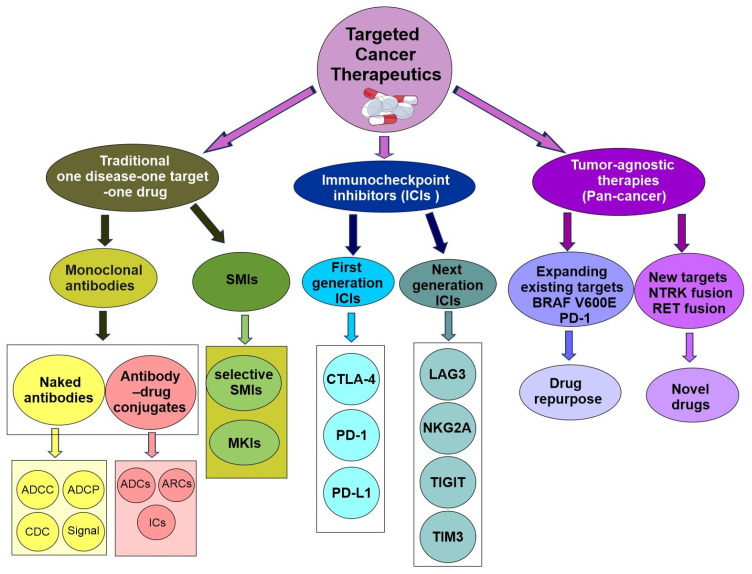
The landscape of current and emerging targeted cancer therapeutics in precision oncology.

The current strategies for developing targeted cancer therapies can be classified into 3 broad categories: the one disease-one target-one drug approach, the systematic targeting immune inhibition approach, and the Pan-Cancer approach (Tumor-Agnostic Therapies) (Figure 6).

#### 5.2.1. The One Disease-One Target-One Drug Approach

The one disease-one target-one drug approach was the initial strategy for targeted cancer therapies in precision oncology. There are two main categories of drugs developed in this approach: antibodies and small molecules (Figure 6). In the initial phase of targeted therapy for cancers, antibodies and small molecular inhibitors were designed based on the identification of cancer-driving oncoproteins for each specific cancer type.

##### MAbs (mAbs)

Köhler and Milstein developed hybridoma technology for producing mAbs in the 1970s. At first, these mAbs were derived from mice. Subsequently, the mouse antibody was gradually humanized to avoid an undesired immune response and to better suit its clinical application. Eventually, 4 types of mAbs became available: murine, chimeric, humanized, and human monoclonal antibodies, which differ from each other by the percentage of murine protein portion present in the immunoglobulin [267]. While the initial antibodies were all naked antibody based on the nature properties of IgG, the engineered antibodies and antibody-drug conjugates have emerged as more effective alternatives.

A very successful example is the development of trastuzumab, an antibody targeting cell surface receptor HER2 to treat HER2-positive breast cancer. In 1987, Slamon et al. identified HER2 gene amplification in roughly 30% of breast cancer tumors, which correlated with poor prognosis [268]. Hudziak et al. developed the HER2-specific monoclonal antibody 4D5 in 1989, demonstrating antiproliferative effects on HER2-positive cells [269]. The antibody was humanized in 1992 for clinical use, becoming Trastuzumab, which showed substantial benefits for HER2-positive breast cancer patients and received approval in the USA (1998) and Europe (2000).

The first monoclonal antibody approved for cancer treatment was Rituximab in 1997 for use in relapsed/refractory indolent non-Hodgkin lymphomas (NHLs). Rituximab, a hybrid antibody, has specific affinity for the B-lymphocyte transmembrane protein, CD20, which is expressed on most malignant B cells [270].

Following the approval of the above first two antibodies, many more mAbs have been developed and approved for the treatment of various cancers. Until 2024, there have been 84 cancer therapeutic antibodies approved by the FDA. Tumor antigens that have been successfully targeted include epidermal growth factor receptor (EGFR), ERBB2, vascular endothelial growth factor (VEGF), cytotoxic T lymphocyte-associated antigen 4 (CTLA4), CD20, CD30 and CD52 [2]. The therapeutic antibodies generated in the early stages are all naked antibodies that utilize its natural properties, including ADCC, ADCP, CDC, and the inhibition of cell signaling.

Despite its huge success, treatment using mAbs alone is often insufficient, and it is often less lethal against cancer cells compared to chemotherapy. Accordingly, the antibody–drug conjugate (ADC) was introduced as an innovative strategy to leverage the specific targeting ability of mAbs with the high potency of cytotoxic agents. An ADC is composed of a tumor-specific mAb chemically linked to a cytotoxic payload via a carefully engineered linker [2,271,272]. Since the first ADC Mylotarg^®^ (gemtuzumab ozogamicin) was approved in 2000 by the FDA [273], there have been 15 ADCs which received market approval by the FDA including two newly approved in 2025. Moreover, over 100 ADC candidates are in the clinical stages of testing for future use. In addition, antibodies have also been conjugated with cytokines (Immunocytokines, ICs) and radionuclide (ARCs). This kind of anti-cancer drug, known as “biological missiles”, is leading a new era of targeted cancer therapy [272] (Figure 6).

##### Small Molecular Inhibitors (SMIs)

It is well established that various gene mutations and protein modifications cause oncogenic alteration of key signaling pathways, which lead to the cancer development. Targeting these oncogenic signaling pathways is thus an effective approach to treat cancer. Besides therapeutic monoclonal antibodies, this approach has led to the development of SMIs able to selectively bind to molecular targets, mostly protein kinases, present in the tumor cells.

There are two types of SMIs based on their selectivity: selective SMIs that are highly selective with single or fewer targets and multikinase inhibitors (MKIs) that have low specificity and exert their anticancer activity by simultaneously targeting a broad spectrum of the human kinases [266] (Figure 6). Selective small molecule inhibitors can be further categorized into selective small molecule kinase inhibitors and selective small molecule nonkinase inhibitors, depending on whether the substrate is a protein kinase [274].

The landmark event in the revolution of targeted therapy was the development in the early ‘90s of the first multikinase inhibitor, Imatinib mesylate. This small molecule inhibitor targets multiple tyrosine kinases such as CSF1R, ABL, c-KIT, FLT3, and PDGFR-β. Specifically, it is used for chronic myelogenous leukemia (CML) and acute lymphocytic leukemia (ALL). Imatinib was approved for medical use in the United States in 2001. It is on the World Health Organization’s List of Essential Medicines.

Gefitinib represents the first FDA approved selective small molecular inhibitor tar-getting EGFR. Gefitinib interacts with the EGFR ATP-binding site and is able to inhibit the abnormal activation of MAPK and PI3K/AKT pathways overexpressed in cancer cells. In early groundbreaking studies, only non-small cell long cancer patients with EGFR mutations were found to be responsive to gefitinib.

Among the selective serine/threonine kinase inhibitors, BRAF inhibitors (Vemuraf-enib and Dabrafenib) and MEK inhibitors (Trametinib and Cobimetinib) are widely used in clinical practice for the treatment of mutated BRAFV600E melanomas, providing significant improvement in survival rates. 

Selective small molecule inhibitors typically interact with a single molecular target, thereby suppressing target-specific cell signaling pathways. Certain cancers are highly dependent on specific dysfunctions affecting processes such as proliferation, survival, apoptosis, differentiation, metabolism, and immune modulation. By antagonizing these critical targets, selective small molecule inhibitors can inhibit aberrant functions or restore normal activity, offering therapeutic benefits in tumor management. Patients undergoing treatment with these agents usually require rigorous screening for the presence or absence of defined genetic alterations, as identified in solid tumor tissue, circulating tumor cells, or other body fluids. Under these circumstances, selective small molecule inhibitors enable effective tumor targeting while minimizing adverse effects associated with off-target inhibition.

Most small molecule inhibitors belong to protein kinase inhibitors. However, drugs involved in DNA repair, epigenetics, apoptosis, tumor metabolism, and beyond are also being discovered. Recently, targets previously viewed as undruggable or difficult to target, such as RAS, have also received approval. Small molecule inhibitors continue to face ongoing challenges, including low response rates and the development of drug resistance.

To date, there are more than 80 FDA-approved small molecular inhibitors. Among the approved drugs, 43 inhibit receptor protein-tyrosine kinases, 20 blocks nonreceptor protein-tyrosine kinases, and 13 target protein-serine/threonine protein kinases are directed against dual specificity protein kinases (MEK1/2). The data indicate that 69 of these drugs are prescribed for the treatment of neoplasms [274,275].

#### 5.2.2. Immune Checkpoint Inhibitors

The 2018 Nobel Prize in Physiology or Medicine was jointly awarded to James P. Allison and Tasuku Honjo “for their discovery of cancer therapy by inhibition of negative immune regulation”. This highlights the prominence and significance of immune checkpoint inhibitors (ICIs) in cancer therapy.

Besides targeting cancer driving molecules within the cancer cells, it is realized that a dynamic crosstalk exists between the immune system and tumor cells which regulates immune evasion, immunoediting, immunosuppression, and immunosurveillance in the TME. Thus, novel research has focused on engaging components of the adaptive immune system. Immune checkpoints engage when proteins on the surface of immune cells called T cells recognize and bind to partner proteins on other cells, including tumor cells. These proteins are called immune checkpoint proteins. When the checkpoint and partner proteins bind together, they send an “off” signal to the T cells. This in part accounts for the aggressiveness of many tumor types including solid and hematologic cancers and explains their ability to evade the immune system [276].

Cytotoxic T lymphocyte-associated protein 4 (CTLA-4) was first identified in 1991 as a second receptor for the T cell co-stimulation ligand B7 [277,278]. Following the identification that the function of CTLA-4 and B7 is to suppress T-cell activation, it was shown in 1996 that anti-CTLA-4 antibodies could clear carcinomas and fibrosarcomas from mice, which provided the first in vivo evidence of efficacy for a checkpoint inhibitor as an anti-cancer therapy [278]. The engineered antibodies targeting CTLA-4, Yervoy (ipilimumab), was approved by the FDA for the treatment of metastatic melanoma in 2011, which marked the first FDA approved ICI and started the rapid expansion of ICI for cancer immune therapy.

Following this, engineered antibodies against programmed cell death-1 (PD-1) and its ligand (PD-L1) immediately gained FDA approval for use against multiple cancer types because of their effect on patient survival. CTLA-4, PD-1, and PD-L1 are first generation targets of ICIs (Figure 6). These discoveries were followed by a significant rise in the identification of novel immune checkpoint targets including LAG3, NKG2A, TIGIT, and TIM3. ICIs targeting these next-generation targets have been developed and many of them are on different stages of clinical trials (Figure 6).

To date, 11 ICIs has been approved by FDA to target T cells via CTLA4 (ipilimumab (Yervoy^®^)) or PD1 (cemiplimab-rwlc (Libtayo), nivolumab (Opdivo^®^), and pembrolizumab (Key-truda^®^)), and target cancer cells and antigen-presenting cell in the tumor microenvironment via PDL1 (and durvalumab (Imfinzi^®^), avelumab (Bavencio^®^), and atezolizumab (Tecentriq)). These ICIs are approved for the treatment of many tumors including breast cancer, bladder cancer, cervical cancer, colon cancer, head and neck cancer, Hodgkin lymphoma, liver cancer, lung cancer, renal cell cancer (a type of kidney cancer), skin cancer, melanoma, stomach cancer, and rectal cancer. Currently, many ICIs are in clinical trials including 13 antibodies to first-generation targets CTLA4, PD1, and PDL1, as well as four antibodies to next-generation targets LAG3, NKG2A, TIGIT, and TIM3 [2,279].

The new progress in the field includes the development of biAbs that simultaneously engage two different immune checkpoints, such as PD1 × LAG3, PD1 × CTLA4, or PDL1 × CTLA4. Combining two ICIs in one biAb as opposed to a mixture of two individual mAbs has cost-saving potential. Moreover, small molecular inhibitors targeting immune checkpoints are also actively explored. Small molecular inhibitors interact with immune checkpoints through multiple mechanisms, such as blocking signaling between tumorigenic factors, promoting immune tolerance, and inhibiting immune inhibitors via epigenetic repression. Many small molecular inhibitors are on various stages of clinical trials [276].

#### 5.2.3. Tumor-Agnostic Therapies, Also Known as Pan-Cancer, or Histology-Independent Therapies

At the beginning, targeted therapy was focused on the development of a series of drugs, each of which was intended to treat a single tumor type with a single molecular aberration. Tumor-agnostic therapies, also known as pan-tumor or histology-independent therapies, are a relatively new approach in cancer treatment that focuses on targeting specific genetic mutations or alterations that drive tumor growth rather than treating tumors based on their location or tissue of origin. The concept behind tumor-agnostic therapies is that some genetic mutations can be drivers of cancer development and progression, irrespective of where the tumor is in the body.

The above discussed ICIs could be considered the earliest tumor-agnostic therapies. In 2017, the FDA expanded the application of Keytruda (pembrolizumab)—which blocks PD-1—to include any metastatic or nonremovable solid tumor exhibiting microsatellite instability-high (MSI-H) or mismatch repair deficiency (dMMR). This was the FDA’s first tissue/site-agnostic approval, meaning that the criteria for patient eligibility are based not on the location of the tumor, but on those molecular characteristics. The field of tumor-agnostic therapy was really set in motion with this landmark FDA approval.

Subsequently, Vitrakvi^®^ (Larotrectinib), an NTRK inhibitor targeting the NTRK gene fusions found in multiple cancers, received accelerated FDA approval in 2018. The drug functions as a tyrosine kinase inhibitor, blocking TRK proteins to prevent cancer cell growth and induce cell death. Specifically, Vitrakvi^®^ was approved to treat adults and children with certain solid tumors that have spread or cannot be removed by surgery and have the NTRK gene fusion. This is another example of the pan-tumor targeted therapy approved by the FDA, which further underscored the potential of tumor-agnostic therapies. In the following years, more tumor-agnostic therapies were approved by the FDA, including targeted therapies (BRAF V600E, RET fusion), immunotherapies (tumor mutational burden ≥10 mutations per megabase, dMMR) and an antibody-drug conjugate (Her2-positive-immunohistochemistry 3+ expression) with pan-cancer efficacy [280].

While the drugs targeting PD-1 BRAF V600E and RET fusion for pan-cancer treatment is considered as an expansion of the existing drugs for pan-cancer, Vitrakvi^®^ targeting NTRK is a drug truly developed for pan-cancer treatment (Figure 6).

Hand in hand with pan-cancer molecular stratification, pan-cancer drug development has evolved rapidly. A recent study performed a pan-cancer analysis of antibody-drug conjugate targets and putative predictors of treatment response [281]. In this study, 121 ADCs clinical trials with 54 targets in 31 cancer types were analyzed and compared with corresponding normal tissues. The analysis indicated that certain ADC are most suitable for a subgroup of cancer types and co-expression of multiple targets was common, which suggests opportunities for pan-cancer therapy with ADC combinations.

Moreover, pan-cancer proteogenomics conducted by the Clinical Proteomic Tumor Analysis Consortium (CPTAC) significantly expanded the landscape of therapeutic targets. The study analyzed over 1000 prospectively collected, treatment-naive primary tumors spanning 10 cancer types, many with matched normal adjacent tissues. By integrating this dataset with other public datasets, this study provides insights into existing cancer drug targets and systematically identifies candidate new targets for drug repurposing or development [282].

Recent pan-cancer analysis identifies CD155 as a promising target for CAR-T cell therapy [283], and identifies MUC1 as a pan-cancer target for drug development for a diverse array of solid tumors and hematological malignancies [284]. Moreover, a recent study explores the possibility to include novel non-protein molecules as pan-cancer targets. They suggest that high mannose (Man 9) oligosaccharides and phosphatidylserine (PS) as non-protein targets for CAR-T therapy [285].

## 6. Cancer Prevention

While cancer cannot be completely prevented, cancer prevention is key to reducing cancer risk. Cancer prevention involves characterizing the prevalence of cancer, identifying etiological factors, and systematically assessing and implementing preventive interventions. Traditionally, research in cancer prevention has emphasized reducing both the incidence of cancer and the mortality associated with the disease.

**Figure 7:**
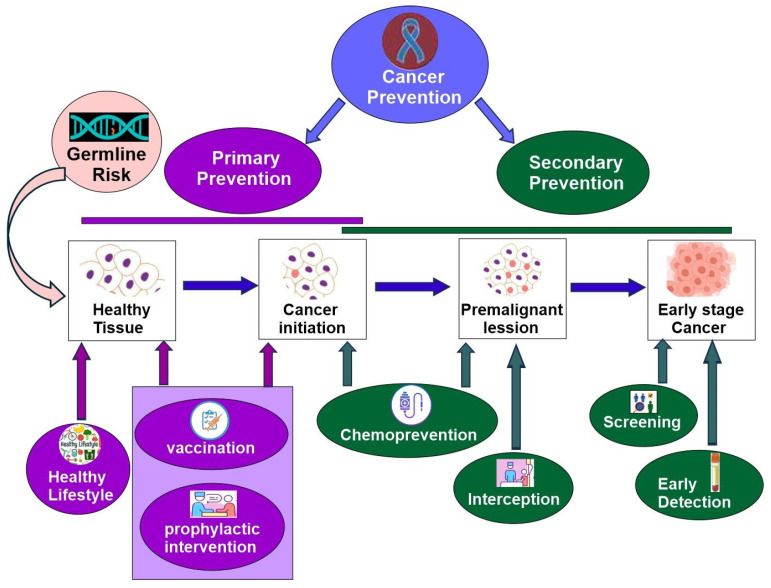
Cancer prevention strategies in precision oncology.

Cancer risk depends on both genetics and environmental factors like behavior, lifestyle, and environmental exposure. Prevention is possible at all stages of cancer development. Multiple strategies for prevention and early detection have been established to decrease cancer incidence, encompassing primary, secondary, and tertiary levels of intervention [4,286]. In general, cancer prevention strategies are classified into primary prevention and secondary prevention based on the timing of the intervention (Figure 7). Other strategies to prevent cancer progression from early stages to metastasis could be instead considered as cancer treatment.

### 6.1. Primary Prevention by Vaccination and Prophylactic Intervention

The aim of primary prevention is to reduce cancer incidence by targeting the general population and people with germline risk. This can be achieved through medical interventions, reducing environmental exposures, and lifestyle changes. Medical intervention includes vaccination and other prophylactic interventions (Figure 7). Some examples are prevention of cervical cancer by human papillomavirus (HPV) vaccination and prevention of liver cancer by vaccination against hepatitis B virus. However, only limited types of cancer are caused by viruses and can be potentially prevented by vaccines. To broaden the application of vaccines, tumor-specific neo-antigens are explored for preventive vaccination with several ongoing clinical trials [4].

Prophylactic intervention is employed following identification of high-risk individuals, such as risk-reducing mastectomy for carriers of BRCA1 or BRCA2 mutations [4]. For other cancer-predisposition syndromes, the focus of management remains on early detection. High-throughput sequencing is facilitating broader access to germline testing in the clinic, resulting in vast amounts of information concerning genetic variants in the population, leading to a refinement of prevention [287,288,289,290,291]. Lifestyle changes include alterations in physical activity or diet, tobacco cessation, or the use of sunscreen, which reduces one’s exposure to environmental carcinogens [4,286].

### 6.2. Secondary Prevention

Secondary prevention strategies include chemoprevention, interception, screening, and early detection, which usually targets premalignant lesions (Figure 7).

#### 6.2.1. Chemoprevention

The broad use of medication to prevent disease is a common strategy used in cancer and other diseases. It is shown that tamoxifen reduces breast cancer incidence in women deemed to be at high risk of cancer development by a third, even after treatment cessation [292,293]. A more recent study showed a relative risk reduction in breast cancer incidence of 65% [294] and 49% [295,296] after treatment with exemestane and anastrozole, respectively. Due to the link between inflammation and cancer development, non-steroidal anti-inflammatory drugs (NSAIDs) and COX2 selective inhibitors (COXIBs), have been utilized as chemopreventive agents. Aspirin has been shown to reduce the incidence of distant metastasis and increase survival in CRC [297,298,299].

However, adverse effects associated with exposure to tamoxifen (endometrial cancers and venous thromboembolism) and aromatase inhibitors (bone fractures) have restricted uptake of chemoprevention drugs outside clinical trials [300]. To overcome the side effects of cancer preventive chemicals, current studies focus on the phytochemical products for cancer chemoprevention. A recent study identified 22 phenolic components from M. citrine fruit extract (MCE) using LC-ESI-MS/MS, which may act as potential future therapeutic agents for cancer prevention [301]. The other compounds include Polyphenol-60 from Green Tea for BC prevention [302], glutelin hydrolysate from riceberry bran residues for the prevention of liver and colon carcinogenesis, phenolic-rich plant extracts from *Lippia citriodora* and *Olea europaea* for pancreatic cancer prevention [303], anthocyanins from various berries for colorectal cancer prevention [304], and Tacrinocerins, which are Tacrine Hybrids with α-Onocerin from *Phlegmariurus*, for lymphoblastic leukemia [305]. In the long run, new, more effective chemopreventive agents with fewer side effects need to be developed before chemoprevention can be more widely accepted.

#### 6.2.2. Interception

Interception lies at the interface of cancer prevention and early detection. Instead of just treating malignancies, Janssen scientists hope to find and eliminate potentially dangerous precancerous cells or disease-driving biological conditions to intercept the disease process and prevent a cancer from ever forming.

For a successful cancer interception, it is essential to understand what drives the progression from premalignancy to malignancy. As discussed above, with the rapid advancement of modern technologies, the efforts through precision oncology have identified many important mechanisms underlying cancer initiation [4,152], which provides a foundation for the development of cancer interception methods.

Indeed, some significant progress has been made. Clinically, interception is already well-established using “mechanical” means including the removal of benign adenomatous colon polyps at screening colonoscopy for colorectal cancer interception, detection and removal of cervical intraepithelial neoplasia (CIN3) at colposcopy for cervical cancer interception, and risk-reducing bilateral sapling-oophorectomy for ovary cancer [306]. In addition, medical interception has also been successfully applied for some cancers. For example, in individuals with von Hippel–Lindau Disease treatment with Belzutifan (an oral hypoxia-inducible factor 2α inhibitor) effectively intercepts the growth of renal cell carcinoma and other tumors by blocking a key biological pathway in oncogenesis [307]. Immuno-interception is also explored as strategy, which eliminate neoplastic lesions at their earliest stages by mobilizing a specific immune response.

With advances in biological insights and precision technology, it is likely that in the future, individuals could be optimally risk-stratified based on their genetics, lifestyle, environmental exposure, and/or other clinical findings. On the basis of this risk stratification, individuals could be intermittently treated with biologically informed interception interventions and monitored by measuring appropriate biomarkers and optimal early detection.

#### 6.2.3. Cancer Screening

Cancer screening and early detection has also been considered as secondary prevention of cancers. The goal of cancer screening and early detection is to cure cancer by detecting premalignant lesions or malignancy prior to the onset of symptoms, which offers the most effective time to treat cancer [308]. Cancer screening significantly contributed to the 25% decrease in the overall cancer mortality rate over the last 100 years. The decline in mortality rate is particularly significant for some broadly screened cancers including breast cancer and colorectal cancer, both of which have a vigorous screening program in place.

The Wilson criteria for disease screening with 9 guiding principles were proposed in 1968 by WHO [309] (Table 1).

Based on the cancer type, screening methods can be either direct or indirect. The direct method is to directly visualize and access the target organ as in colorectal and cervical cancer. Indirect methods rely on the measurement of biomarkers associated with cancer (e.g., CA-125 or PSA for prostate cancer), or radiographic imaging (e.g., mammography for breast cancer).

Due to tumor heterogeneity, indirect methods of cancer screening frequently lead to compromised screening efficacy due a decrease in performance characteristics of the screening technique and an increase in overdiagnosis and overtreatment [310]. Ideally, cancer screening is undertaken when the risk of cancer is high enough to justify the risk of overdiagnosis and overtreatment in an otherwise healthy population.

Through the evaluation of outcomes associated with commonly screened cancer types—including breast, prostate, cervical, colorectal, and lung cancers—several key insights have emerged: (1) invasive cancers exhibit a spectrum of biological behaviour, ranging from indolent to highly aggressive forms; (2) the majority of precancerous lesions are not obligatory precursors to invasive cancers; (3) effective screening programs that target precursor lesions or early-stage malignancies should result in a corresponding decrease in advanced-stage cancer incidence; and (4) the benefits of screening are not uniformly distributed across all individuals.

Learning from these lessons, a recent article provided corresponding improvements by incorporating key clinical questions at each step of the screening ‘cascade’ proposed by Harris et al. [311]. Based on clinical characteristics and molecular profiles of detected cancers, the new model is designed to enable precision screening through individualized risk prediction [310]. Specifically, screening decisions should take into account a person’s pretest probability of cancer, the threshold risk level where testing would provide net benefit, and patient perspectives regarding risk tolerance. The screen should mitigate overdiagnosis by testing strategies that lower the chance of detecting unimportant lesions.

Overall, due to the heterogeneous nature of cancers, the screening strategy has to be tailored for each cancer type with the consideration of the unique characters of each population group. The knowledge regarding who is at risk of which cancers, in terms of both site and biology, is critical and needed to improve cancer screening.

#### 6.2.4. Early Detection

The aim of early detection is to reduce the proportion of patients diagnosed with cancer at a late stage to maximize the probability of cure.

The above discussed cancer screening is a major approach for cancer early detection. For many cancers, such as lung, breast, cervical, and colorectal cancers, this is a crucial aspect of cancer control. Many studies have demonstrated the value of cancer screening in lowering the mortality rate. Between 2013 and 2017 in the UK, 1-year net survival rates for CRC were 97.7% at stage 1 versus 43.9% at stage 4; for lung cancer, 87.7% at stage 1 and 19.3% at stage 4; and for breast cancer, 100% at stage 1 and 66% at stage 4 (Office for National Statistics, 2019). Screening programs have been shown to reduce mortality in many types of cancers [4,312,313,314,315,316]. Many assays and platforms have been established to employ ctDNA technology in early cancer detection. These assays and platforms include CancerSEEK, DETECT-A, Galleri assay, whole-genome sequencing for copy number variation (WGS-CNV), and whole-genome bisulfite sequencing (WGBS) [98,317,318,319,320].

A recent study with a targeted multi-cancer early detection (MCED) approach evaluated more than 100,000 informative methylation regions among 6689 participants (including 2482 cancer patients in more than 50 cancer types). The study showed high sensitivity in detecting cancers in its early stages [321]. Beyond methylation profiling, fragmentomics and topological analyses are alternative approaches to ctDNA analysis [322,323].

AI has also been introduced to enhance the analysis of huge ctDNA databases to increase the sensitivity and specificity for early cancer detection. In a recent study, a bio-hybrid platform comprising trained detection canines and AI tools was employed to detect breast, lung, prostate, and colorectal cancer in breath samples of 1386 participants. The results demonstrated high sensitivity and specificity and enables early-stage cancer detection [324].

The promise of ctDNA assays in early cancer detection may be further enhanced by our understanding of ctDNA kinetics and the relationship of ctDNA fraction that may complement conventional pathological tumor staging. This understanding may identify patients with tumors that are more likely to recur following surgical intervention and in whom neoadjuvant and adjuvant treatment can be tailored accordingly.

## 7. Cancer Diagnosis

Once cancer is suspected, a definitive diagnosis typically requires histopathology. These techniques not only confirm malignancy but also classify cancer by subtype, grade, and stage.

### 7.1. History

In ancient times, the diagnosis of cancer was rudimentary, primarily based on visual and palpable observations. The advent of the microscope in the 17th century allowed scientists to examine cells and tissues more closely, leading to the discovery of certain cancerous cells. By the 19th century, advancements in histopathology and the establishment of laboratory medicine significantly improved the accuracy of cancer diagnoses.

In modern time, cancer diagnosis usually involves personal and family medical history, physical examination, lab testing, imaging, and biopsy, the last of which is often the only and final test to either confirm or deny a cancer diagnosis. These tests often follow a positive screening test or a symptom.

Some common types of lab tests used to help diagnose cancer include blood chemistry tests, complete blood count (CBC), cytogenetic analysis, immunophenotyping, liquid biopsy, sputum cytology, tumor marker tests, urinalysis, and urine cytology. Imaging tests used in cancer diagnosis include CT, MRI, nuclear scan, bone scan, PET scan, ultrasound, and X-rays.

The traditional biopsy is a procedure to remove a piece of tissue or a sample of cells from the body so that it can be tested in a laboratory. Types of tissue biopsy include needle biopsy, endoscopic biopsy, skin biopsy, bone marrow biopsy, and surgical biopsy. These biopsies are all tissue-based invasive biopsy. Tissue biopsy has been the gold standard in cancer diagnosis, which allows for the typing and grading of the tumor cells and the identification of target expression for targeted therapies.

Tissue biopsies, typically performed under image guidance, are specialized invasive procedures associated with notable morbidity and financial considerations. These interventions often require patients to attend tertiary care centers equipped with advanced facilities. Beyond logistical challenges, several factors can limit tumor tissue acquisition, including difficulty in accessing the lesion, proximity of the tumor to critical organs or vascular structures, patient comorbidities, and patient hesitancy due to procedural risks. In many cases, repeat biopsies are clinically indicated.

As the world enters the era of precision oncology, cancer diagnosis tools have been modernized significantly to improve the accuracy of diagnosis and are more convenient for patients with lower costs and higher efficiency. In particular, non-invasive biopsies have made great advances.

### 7.2. Cancer Diagnosis by Liquid Biopsy

We have discussed the role of liquid biopsy in the early detection of cancer. Recently, CTCs have emerged as a compelling reservoir of biomarkers with profound implications for cancer diagnosis, prognosis, and recurrence prediction. For cancer diagnostics, the identification of CTCs in the bloodstream holds the potential for signaling the presence of a primary tumor. CTCs offer a distinctive advantage as they represent intact tumor cells released from primary or metastatic tumors. This unique characteristic allows CTCs to be used as a source of comprehensive information across various molecular levels, encompassing DNA, RNA, protein, and metabolites. Therefore, analysis of CTC provides more comprehensive and accurate early diagnosis of cancer. CTC monitoring uses routine blood drawings, making it less invasive and more convenient than tissue biopsies, and offers an easier way to detect and track cancer progression.

### 7.3. Diagnosis with Molecular Imaging

Cancer molecular imaging is the non-invasive visualization of molecular and cellular processes characteristic to neoplasia, such as proliferation, glucose metabolism, and re-captor expression. Their inherent characteristic of visualization of malignant cells has the potential to enhance cancer diagnosis and staging on multiple levels [325]. In general, molecular imaging plays a vital role in cancer diagnosis by enabling early detection of cancer at the molecular level, accurate staging and assessment of tumor extent, identification of specific molecular targets for therapy, and monitoring of treatment response and disease progression.

Molecular imaging applications can make properties of carcinogenesis visible at much earlier time points because alterations on the cellular level are targeted and can potentially be detected as soon as they occur. For example, abnormalities in malignant cells’ glucose metabolism occur at very early time points in carcinogenesis [326]. While differentiation between benign and malignant tumors on conventional CT and MRI can be difficult, molecular imaging allows for a much better assessment of tumor because functional properties of malignant cells are visualized.

Incorporation of AI into molecular imaging and radiomics have offered significant opportunities for advancement. Numerous dynamic applications of AI exist, including image interpretation and classification, data organization, information mining, storage, and integration, and much more. AI is anticipated to greatly assist pathologists in enhancing diagnostic specificity because of its broad application in biomedical imaging technology [327,328]

Future directions for molecular imaging in cancer diagnosis include nanotechnology, multimodal imaging, and radiomics and AI. Nanoparticles can target tumor cells and cross cellular membranes, which may lead to highly sensitive imaging agents with therapeutic capabilities. Combining various imaging techniques, such as PET/MRI or PET/CT, provides more detailed multi-layer information, which may become standard in cancer diagnosis. Moreover, incorporation of AI into radiomics will enhance diagnostic accuracy and sensitivity.

Simultaneously, progress in imaging equipment, innovative algorithms, and AI has opened avenues to increase the efficiency and accuracy of cancer diagnosis.

## 8. Clinical Implementation Challenges

Precision oncology is a major advancement in cancer care with lasting impact. Despite its promise, adoption is limited by complex data management needs, high costs, access disparities, ethical concerns, tumor heterogeneity, lack of diverse genetic data, and gaps in clinician education.

### 8.1. Challenges to Clinical Implementation of Precision Oncology

Technologies like next-generation sequencing, multigene assays, and companion diagnostics are now commonly used to detect actionable mutations and guide therapy choices for cancer patients [329]. Clinical practice guidelines, such as those from the National Comprehensive Cancer Network, recommend comprehensive biomarker testing for most patients with advanced or metastatic cancer to guide treatment decisions [330]. Despite these recommendations, emerging molecular testing is not fully adopted. Barriers are especially significant in community oncology settings—which serve most North American cancer patients—including delays and limitations in tissue access, insufficient infrastructure for testing, and challenges with sample handling and ordering [331,332]. Although individualized treatment strategies based on precision oncology analysis that considers molecular profiling and other clinical testing data are technically, logistically, and financially achievable, this approach is not comprehensively implemented currently [333].

More targeted therapies are gaining approval based on genomic biomarkers across different cancers. Some of these drugs show effectiveness in multiple tumor types with shared molecular changes, leading to broad or tumor-agnostic approvals. Each year, new targeted treatments for biomarker-defined patient groups are approved, but this creates major challenges for healthcare systems to update infrastructure and reimbursement policies to ensure patient access. A major gap exists between anticancer drug progress and patient access, which may worsen health disparities due to unequal technology access and limited clinical implementation knowledge [330]. A recent large-scale study showed that about 23% of patients with newly diagnosed advanced NSCLC did not undergo genomic testing for any of the four guideline-recommended targets (ALK, BRAF, EGFR, ROS1) before first-line treatment, illustrating how delays in biomarker testing hinder access to new therapies [334].

### 8.2. Regulatory Challenges in Precision Oncology

The regulatory pathway for precision medicine is an evolving and adaptive process that involves early engagement with agencies, selecting a path based on device classification and intended use, and utilizing innovative regulatory tools like accelerated approval. The regulatory framework for precision medicine is still evolving to keep pace with rapid innovation in areas like genomics, artificial intelligence, and manufacturing. The key challenges in precision oncology regulation include but not limited to the following areas.

(1) Small patient populations. Small patient population often resulted in fewer participants in the clinical trials and thus make it difficult to assess value using traditional methods. (2) Rapid Innovation. The rapid advancement in technologies challenges existing assessment processes. (3) Complex data interpretation and integration. This includes ensuring the privacy and security of large-scale genomic databases need to be protected. It is difficult to interpret huge data from diverse source and integrate them into routine care. In addition, AI/machine learning algorithms used in healthcare need to be validated. (4) Health system strain due to increased demand on healthcare infrastructure and required changes in professional roles. (5) Global harmonization. Differences in regulatory requirements across countries can create hurdles for drug and diagnostic developers. (6) Advanced manufacturing. Personalized therapies, especially cell and gene therapies, involve complex manufacturing processes that require novel regulatory approaches to ensure quality and safety.

The FDA manages personalized medicine products through three centers: CDER, CDRH, and CBER. While each enforces long-standing regulations, these rules often fall short in handling the complexities of personalized medicine, where product interdependence affects safety and effectiveness. Consequently, there are inconsistencies in the regulation of personalized medicine products [335].

To overcome the challenges, we need to fully understand and explore various regulatory pathways to obtain early regulatory approval for various clinical applications of precision cancer management. This include early engagement by initiating discussions with regulatory bodies like the FDA during the early stages of product development, which is crucial for tailoring a strategy that aligns with business goals and meets requirements. We also need to explore various regulatory approaches such as accelerated approval and conditional approval. Data and evidence need to be provided continuously and in a timely manner. There continues to be a need for high quality evidence that precision medicine actually improves patient outcomes if it is to be widely adopted. When the “gold standard” of large randomized clinical trials is infeasible, alternatives creative approaches such as new models of risk-sharing and evidence development between technology developers, health care systems, and payers should be adopted to generating evidence. Strong evidence on how precision medicine affects patient outcomes will support approval and reimbursement [336].

### 8.3. Ethical Issues in Precision Oncology

Ethical issues in precision medicine include privacy and data security, the risk of genetic discrimination, challenges in obtaining truly informed consent due to complex information, ensuring equitable access to costly personalized treatments, potential negative psychological impacts, maintaining the doctor-patient relationship, and addressing scientific concerns like incidental findings and evidence gaps [337,338].

Large-scale collection of sensitive health and genetic data creates risks for breaches and misuse. Data breaches in electronic health record platforms potentially reveal sensitive personal and health information. Some professionals consider apprehension about data security not being guaranteed [337]. According to certain researchers, the primary risk stems from inadequate data handling practices, such as individuals transferring sensitive information to personal devices like laptops or flash drives [339]. Patients concern about the hacking of online data [340]. Determining who can access this data (employers, insurers, researchers) and under what conditions is a major concern. The confidentiality of genetic information to families rather than to individuals is also an issue under debating, although “Familial approach to confidentiality” is conceptualized in UK genetic guidelines [341]. Moreover, genetic testing can reveal unexpected information about predispositions to other conditions, creating ethical dilemmas about what to tell patients and their relatives.

Concerns about informed consent have also emerged as a problem for precision medicine. Patients may struggle to understand the full implications of complex genetic tests and personalized treatment options. Specific informed consent concerns include the medical field’s limited ability to clinically address genomic testing results, identifying appropriate detail levels in consent processes [342], introducing new consent formats, and managing patients’ expectations regarding genomic testing [343]. In addition to the permission issue, easy access to the genetic information of people by third parties such as insurers raises questions regarding discrimination. The findings of genetic tests may be used as grounds for discrimination by health and life insurance companies. As of right now, 47 nations have enacted antidiscrimination insurance plans based on genetic information [344]. Moreover, genetic testing can reveal unexpected information about predispositions to other conditions, creating ethical dilemmas about what to tell patients and their relatives.

### 8.4. Disparities in Precision Oncology

Precision medicine selects medication according to genetic data, social history, and environmental factors, aiming to tailor treatment for individual patients. However, many disparities shatter this promise for numerous groups. Access to genomic data is limited in high-income countries, and socioeconomic disparities hinder healthcare equality, particularly for low-income individuals or those without insurance. The digital divide, lack of education, and different ethical standards and regulatory frameworks all contribute to disparities in precision cancer treatment [344].

Global genetic testing shows notable gaps and limitations. Geographic representation in genomic data collection is heavily skewed toward Europe [345]. For example, current carrier screening panels, while including over 1000 alleles for more than 200 illnesses, are primarily composed of alleles common to persons of European descent, limiting their utility for people of different ancestries [346].

In addition to genetic testing, the difficulties extend beyond diagnostics to treatment availability. It was shown that 34% of uninsured Americans patients who underwent NGS testing being unable to obtain FDA-approved targeted drugs due to cost barriers [347]. These issues are magnified on a global scale, where developed nations’ emphasis on diseases affecting their populations, combined with their greater financial resources for sophisticated medical interventions, perpetuates a cycle in which genetic research primarily benefits already-privileged populations, exacerbating existing healthcare disparities [348].

Precision medicine also faces several critical gender specific disparities that require attention. Current social and economic factors influencing women’s access to medical treatment may also affect their ability to obtain precision medicine. The gender disparities are notably more severe in regions where women face additional barriers to medical access [349].

### 8.5. Potential Solutions

Addressing these challenges will require a multi-faceted approach, including: expanding research infrastructure to build high-quality, diverse genomic datasets, promoting partnerships between academic institutions, industry, and healthcare providers to develop and implement scalable solutions, investing in education for both healthcare professionals and the public to improve genomic literacy and build trust, developing robust data governance frameworks that protect patient privacy while enabling responsible data sharing, increasing workforce diversity, developing new economic and payment models to break the economic barrier, and reforming clinical trial and regulatory frameworks to better accommodate the specific needs of precision medicine [350].

## 9. Future Perspectives

Despite being the most developed area in precision medicine, precision oncology is still in its early stage. Great efforts in all aspects of precision oncology as outlined in Figure 1 are needed to achieve the precision cancer management for every patient.

In terms of technology and knowledge, while the innovative technologies are still emerging, the focus of the immediate future is to integrate the vast data generated by various methods. We have seen a clear effort in the scientific community to achieve this. AI has rapidly emerged as a transformative force and may play a vital role in this regard. The convergence of AI and precision medicine promises to revolutionize health care. AI leverages sophisticated computation and inference to generate insights, enables the system to reason and learn, and empowers clinician decision making through augmented intelligence [119,351]. As we need to further understand the biology of tumor cells, emerging and future studies will explore intratumor heterogeneity and consider tumor cells and TME as one ecosystem to understand the interactions among tumor and non-tumor cells withing the ecosystem. Cancer driver genes and clonal expansion concepts have advanced our understanding of tumor initiation enormously and will continue to be developed for a complete understanding of the transition of cells from pre-malignant to malignant. This is crucial for early and accurate intervention to prevent cancer.

In terms of the central tenet, pan-cancer analysis has gained momentum recently and may have profound implication in future precision oncology including cancer subtype stratification and targeted treatment. It is possible that cancers can be re-classified not only by cancer cells but also by other cell types in the TME. For example, reclassification of cancer by immune cells subtypes across traditional defined cancer types could maximize the efficacy of immunotherapy [2,226,352]. Accordingly, Tumor-Agnostic Therapies or pan-cancer therapies is rapidly gaining momentum and will be the future targeted cancer therapies

However, clinical implication of precision oncology is the weakest area and will be the future focus of precision oncology. Despite the rapid development, well-accepted concepts and principles, accumulated data, and acknowledged merits of precision oncology, its full potential for advancing cancer prevention and treatment remains unrealized due to the vast translational challenges. To achieve of the true potential of precision oncology, future studies must focus on overcoming these translation challenges including clinical infrastructure, education of professionals and patients, economic burden, regulation, health care policy, regulatory pathways, and administrative alignment.

## Figures and Tables

**Table 1:** Wilson criteria for disease screening.

1	The condition sought should be an important health problem
2	There should be an accepted treatment for patients with recognized disease
3	Facilities for diagnosis and treatment should be available
4	There should be a recognizable latent or early symptomatic stage
5	There should be a suitable test or examination
6	The test should be acceptable to the population
7	The natural history of the condition, including development from latent to declared disease, should be adequately understood
8	There should be an agreed policy on whom to treat as patients
9	The cost of case-finding (including diagnosis and treatment of patients diagnosed) should be economically balanced in relation to possible expenditure on medical care as a whole
10	Case-finding should be a continuing process and not a “once and for all” project

## Data Availability

No new data were created or analyzed in this study.
